# Structural and spectral properties of generative models for synthetic multilayer air transportation networks

**DOI:** 10.1371/journal.pone.0258666

**Published:** 2021-10-21

**Authors:** Marzena Fügenschuh, Ralucca Gera, José Antonio Méndez-Bermúdez, Andrea Tagarelli

**Affiliations:** 1 Dept. of Mathematics, Physics, and Chemistry, Berliner Hochschule für Technik, Berlin, Germany; 2 Dept. of Applied Mathematics, Naval Postgraduate School, Monterey, CA, United States of America; 3 Instituto de Física, Benemérita Universidad Autónoma de Puebla, Puebla, Mexico; 4 Dept. of Computer Eng., Modeling, Electronics, and Systems Eng. (DIMES), University of Calabria, Rende (CS), Italy; Unviersity of Burgundy, FRANCE

## Abstract

To understand airline transportation networks (ATN) systems we can effectively represent them as multilayer networks, where layers capture different airline companies, the nodes correspond to the airports and the edges to the routes between the airports. We focus our study on the importance of leveraging synthetic generative multilayer models to support the analysis of meaningful patterns in these routes, capturing an ATN’s evolution with an emphasis on measuring its resilience to random or targeted attacks and considering deliberate locations of airports. By resorting to the European ATN and the United States ATN as exemplary references, in this work, we provide a systematic analysis of major existing synthetic generation models for ATNs, specifically ANGEL, STARGEN and BINBALL. Besides a thorough study of the topological aspects of the ATNs created by the three models, our major contribution lays on an unprecedented investigation of their spectral characteristics based on Random Matrix Theory and on their resilience analysis based on both site and bond percolation approaches. Results have shown that ANGEL outperforms STARGEN and BINBALL to better capture the complexity of real-world ATNs by featuring the unique properties of building a multiplex ATN layer by layer and of replicating layers with point-to-point structures alongside hub-spoke formations.

## Introduction

Airline Transportation Networks (ATNs) attracted more attention as they are generally more efficient, safer, and can easily connect remote areas compared to other means of travelling [1]. In particular, network science studies are surfacing primarily on the U.S. airline network, as it is one of the most advanced transportation infrastructures in the world blending services offered by the commercial, military, and public environments [2–4].

To start with, the specific hub and spoke structure of ATNs generated interest in identifying hub locations [3]. More recently, the evolving structure of the ATNs provided valuable data and a need for network analysis research to provide an understanding of their structure and run simulations of their spectral and resilience properties [4]. This growth of the airlines is dynamic, determined not only by the decisions and connections of other carriers but also by economic and political factors worldwide. We model this interconnected world as a multilayer network, the layers of which capture the network of each airline independently, while the entire ensemble then covers the mutual dependency between the airlines [5–8]. This model thus can be analyzed at the layer level or in its entirety as a system, as a more comprehensive extension of the monoplex networks [9–11].

While airline flights modeling as a hub-and-spoke network was introduced in the ‘90s as the primary company’s strategy in organizing their routes, network analysis gained traction in analyzing airlines and their interconnections over the last couple of decades by the air transportation management community [12–15]. As major carriers have switched from linear route structures to hub-and-spoke ones, they compete for the flights from the hubs to the outposts while having a monopoly over the flights from their airline’s hubs [16]. This particular behavior directs our focus on the hub-and-spoke network structure models, generally driven by cost and demand [17], competition and market uncertainty [18], transport momentum and aircraft load factor [19], capacity decisions before demand met [20], the regional jet technology, and the low-cost business model [21], or alliances and mergers in the airline industry [22]. Complementary to network science approaches in modeling the network structure, other approaches use duopoly games [23], airline competition models based on loyalty [24], and “differentiated duopoly model that accounts for congestion externalities, passenger benefits from increased frequency, passenger connecting costs, and airline endogenous hub location” [25].

In order to provide dependable insights into a variety of issues related to the ATNs, one needs multiple data sources concerning, for instance, vulnerability, inter-dependencies of airports and airlines, but also virus propagation, etc. [26–30]. Of great interest is the creation of network models for these ATNs, such as the world air transportation system [27, 31], the U.S. airline transportation system [32], the Brazilian [33], the Indian [34], and the Chinese one [35]. In the last few decades, particular attention has been paid to understanding multilayer ATNs [36–41]. As an example, the Chinese air traffic network has been analyzed and modeled as a multilayer network. One proposal consisted in representing the ATN by three layers, namely the core, bridge, and periphery layers using a *k*-core decomposition of the original network [37]. Additionally, the approach proposed in [42] focuses on the identification of airway, route, and flight layers, the mapping relationships of which are investigated as an integrated multilayer ATN.

Generative modeling is an active network science research area, with a recent emphasis on synthetic multilayer network creation [11, 43–46]. Common approaches of growing multilayer network models are based on the *preferential attachment* model introduced for social networks, with just a few publications focused on creating synthetic multilayer airline transportation networks [5–7, 47].

Our work follows the above mentioned line of research, focusing on the European Air Transportation Network (EATN) [36, 47, 48], which is viewed as a composition of connections within and between different airlines, each being modeled through a layer of the multiplex network. A seminal paper in this type of modeling introduces the BINBALL synthetic model [6]. That is, the layers are initialized with an equal number of nodes, and then edges are added in a preferential attachment manner similar to the other multilayer social networks. A further extension is introduced by the STARGEN model that extends the preferential attachment of BINBALL model by enforcing both a differentiated layer set sizes as well as a hub-spoke layer model observed in the early studies at ATNs [44]. Moreover, a different approach is defined in the ANGEL model, which removes attention from the preferential attachment to gain more influence on the intra- and inter-layer structure of the multilayer synthetic network created [45]. A preliminary analysis and comparison of these synthetic models followed recently [46], which we use as inspiration for this work.

### Contributions

Our goal in this work is an extensive analysis of the three synthetic models, BINBALL, STARGEN, and ANGEL, in terms of topological, resilience, and spectral properties. Although all three models were formulated to mimic the same reference network, they differ in their approaches. One major concern is to demonstrate that the pure preferential attachment approach, which is adopted in BINBALL and STARGEN, is not sufficient to imitate the complex structure of an airline transportation network, especially viewed as a multiplex. By contrast, being designed to generate a multiplex layer by layer and to balance between the number of hub-spoke and point-to-point structured layers, the ANGEL model lends itself as the most sophisticated generative model to effectively mimic a real-world ATN. Experimental results from the various stages of analysis we carried out, have indeed unveiled that only the ANGEL provides reliable approximations in all facets of the complex reference networks, while STARGEN and BINBALL perform comparably mainly on the macroscopic level, i.e., when viewing the entire multiplex.

We compare the synthetic networks to the reference ones also in terms of resilience, in both site and percolation process scenarios, under different types of attack. Besides, we investigate on the failure effects in relation to the presence of both hub-spoke and point-to-point structures, which is a unique feature of the ANGEL model. Moreover, we analyze spectral and eigenfunction properties of the synthetic and reference networks, based on the Random Matrix Theory modeling approach. Even in this evaluation stage, ANGEL turns out to be the best suited model w.r.t. both EATN and USATN.

The remainder of the paper is organized as follows. Section *Background* gives an overview of the three methods under study as well as the reference networks we use for validation. Section *Topological Analysis* encompasses our extensive structural analysis of the synthetic networks generated by the three methods versus the real instances. Section *Resilience Analysis* is devoted to site and bond percolation process to validate the resilience behavior of the synthetic and reference networks, whereas Section *Spectral Analysis* contains our study on the eigenfunction properties of the synthetic and reference networks. Also, an insight into the efficiency of the three generative methods is provided in Section *Running Times*. Section *Discussion* summarizes the key traits of the three methods, highlights their features but also points out their limitations. Finally, in Section *Conclusions*, we summarize our contributions and give an outlook on what our work can be used for and how it could be continued.

## Background

Before we commence our comprehensive study, we provide background information on both the synthetic network generation models and reference network data that we will use in this work.

### Synthetic network generation models

As it is well-known in the complex network literature, a multiplex is a multilayer network, the layer graphs of which are defined on the same set of nodes.

In our setting, layers correspond to different airlines, nodes to airports, and edges to flight connections; this means that two or more flights for the same pair of airports may occur though referring to different airlines. Each node in the multiplex can be considered both from a local and a global perspective: this reflects on the notion of degree, which hence can be defined locally, i.e., the degree of a node within a particular layer graph or globally, i.e., the total degree of a node across all layers.

Let *n*, *m*, and *l* denote the total number of nodes, edges, and layers in the multiplex to be generated. BINBALL initializes the node set *V*_*L*_ in each layer *L* by uniformly distributing the *n* nodes (i.e., by dividing the multiplex node-set *V* into possibly equally-sized subsets). At each iteration, an edge is created and added to a layer selected uniformly at random. To create an edge, the two end-nodes are selected in a preferential attachment manner according to their local and global degrees; more precisely, one end-node *u* is sampled from a probability distribution *f*_*L*_(*V*_*L*_, Θ) that is directly proportional to the local degree of *u*, and the other end-node *v* is sampled from a probability distribution *f*_*M*_(*V*, Θ) that is directly proportional to the global degree of *v*, where Θ denotes a set of parameters that might account for node weighting schemes. We refer to *f*_*L*_(⋅) and *f*_*M*_(⋅) as the local and global preferential attachment functions, respectively. It should be noted that BINBALL produces a multiplex composed of layers with similar sizes for both the node and edge sets. This limitation is overcome in STARGEN, which is designed to differentiate the growth of the layers, allowing for different sizes according to a non-uniform distribution *P*_*edgeL*_ of the layers’ edge counts. Moreover, STARGEN introduces local and global scaling factors in the probability functions *f*_*L*_ and *f*_*M*_, respectively, so to impact on the diversification of the intra-layer topology based on the different weights assigned to each layer. Despite their differences, BINBALL and STARGEN share a common network generation scheme, which is captured in Algorithm 1. Note that we use superscripts (*S*) and (*B*) to distinguish between the preferential attachment functions and parameters used in STARGEN and BINBALL, respectively. We refer the interested reader to [6, 44] for further details on the two models.

**Algorithm 1**: BINBALL and STARGEN models

**Input**: Total number of nodes *n*, edges *m*, and layers *l* desired in the multiplex, distribution *P*_*edgeL*_ (uniform for BINBALL) for the layers’ edge-set sizes

**Output**: Layers *L*_1_, …, *L*_*l*_ and the multiplex *M*

 {*Initialize data structures*}

1: Let *L*_1_, …, *L*_*l*_ be empty graphs representing layers

2: Let *M* be the multiplex with *n* isolated nodes

3: **for** *i* = 1 **to** *m* **do**

4:  Sample layer *L* from *P*_*edgeL*_

5:  Sample node *u* from the local preferential attachment function $f_{L}^{\left(B\right)}\left(V_{L},\Theta^{\left(B\right)}\right)$, resp. $f_{L}^{\left(S\right)}\left(V_{L},\Theta^{\left(S\right)}\right)$

6:  Sample node *v* from the global preferential attachment function $f_{M}^{\left(B\right)}\left(V,\Theta^{\left(B\right)}\right)$, resp. $f_{M}^{\left(S\right)}\left(V,\Theta^{\left(S\right)}\right)$

7:  Add the edge (*u*, *v*) to *L* and *M*

8:  Update local and global degrees of *u* and *v*

9: **end for**

The hub-spoke layers produced by both BINBALL and STARGEN result in homogeneous structures due to the way a preferential attachment method is applied. However, as found in [45] based on a thorough investigation of the EATN network, most layers appear to show a mixture of both hub-spoke and point-to-point structures. Addressing this crucial aspect is a major focus in the ANGEL model, the algorithmic scheme of which is sketched in Algorithm 2.

Initially, ANGEL distributes the nodes of the multiplex among the layers (according to the distribution *P*_*layerN*_), favoring their overlapping (according to the distribution *P*_*nodeL*_). The next stage is the identification of hubs. For this purpose, since hubs are usually found as nodes with a central geographical location, a further notable enhancement introduced in ANGEL is that it incorporates the spatial location of the nodes in the network, by randomly distributing the nodes of a layer on a grid (with a shape proportional to the square root of the node-set size); next, a minimum spanning tree is computed according to the Euclidean distance between the nodes, and eventually used to identify the hubs of that layer. A hub-subnetwork is then formed by using the configuration model with respect to a degree sequence sampled uniformly between 1 and the total hub count in the multiplex.

Unlike BINBALL and STARGEN, which generate all layers simultaneously, ANGEL enables each layer to be formed separately from one another, in such a way that point-to-point structures in the layers are mimicked alongside the hub-spoke structures. For each layer, nodes are assigned to points on a grid and a minimum spanning tree is computed. For the point-to-point strategy, too long and too short distances are avoided while adding a number of edges randomly chosen in the range between one and the difference between the edge-set size of the replicated layer and the edge count of the minimum spanning tree calculated; the remaining edges are added according to a preferential attachment. To create the hub-and-spoke structure of the layer, low degree nodes are iteratively connected with nodes close to the spatial center of the minimum spanning tree; then a certain percentage of edges sorted by increasing distance are chosen, with one end being a hub, and the remaining amount of edges is attached in such a way that low degree nodes but leaves are preferably linked with high degree nodes but hubs. The multiplex finally emerges as a multigraph obtained as the union of all nodes, discounting repetition, and all the edges in the layers, allowing the repetition from different layers. We refer the interested reader to [45] for further details.

**Algorithm 2**: An outline of the ANGEL model

**Input**: Total number of nodes *n*, edges *m*, and layers *l* required in the multiplex, distribution *P*_*edgeL*_ for the layers’ edge-set sizes, distribution *P*_*nodeL*_ of the node count per layer, distribution *P*_*layerN*_ for the random selection of the number of layers a node appears in, and the percentage *p* of layers to be formed by the point-to-point strategy

**Output**: Layers *L*_1_, …, *L*_*l*_ and the multiplex *M*

 {*Initialize data structures*}

1: Let *L*_1_, …, *L*_*l*_ be empty graphs representing layers

2: Let *M* be the multiplex with *n* isolated nodes

 {*Assign nodes to layers*}

3: **for** *u* ∈ *M* **do**

4:  Sample layer repetition count, *r*_*u*_, from the *P*_*layerN*_

5:  Select *r*_*u*_ different layers, according to *P*_*nodeL*_, to place *u* in

6: **end for**

 {*Create hub-subnetwork*}

7: Assign hubs to layers and create a multigraph on all hubs using configuration model

 {*Create layers*}

8: Assign number of edges to layers according to *P*_*edgeL*_

9: **for** *i* = 1 **to** ⌊*p* ⋅ *l*⌋ **do**

10:  Call a point-to-point layer creation procedure for *L*_*i*_

11:  Add all edges from *L*_*i*_ to *M*

12: **end for**

13: **for** *i* = ⌊*p* ⋅ *l*⌋ **to** *l* **do**

14:  Call the hub-spoke layer creation procedure for *L*_*i*_

15:  Add all edges from *L*_*i*_ to *M*

16: **end for**

### Reference networks

The European ATN (EATN) was originally studied in [49] and extensively analyzed in our earlier work [45]. We refer the interested reader to the above works for further details, whereas here we provide an overview through a selection of statistics reported in Table 1.

**Table 1:** Main statistics on the EATN.

	multiplex	layers (# 37)		
		max	min	mean
*#nodes*	417	128	35	54.97
*#edges*	3 588	601	34	96.97
*density*	0.04	0.11	0.03	0.06
*transitivity* (*)	0.30	0.34	0	0.07
*degree*	17.21±27.78	9.39±11.55	1.94±2.54	3.13±6.07
*average path length*	2.76±0.80	3.35±1.43	1.94±0.18	2.25±0.56
*clustering coefficient* (*)	0.42±0.33	0.55±0.47	0±0	0.20±0.28

(*) Values calculated with discarded multiple edges

**Fig 1:**
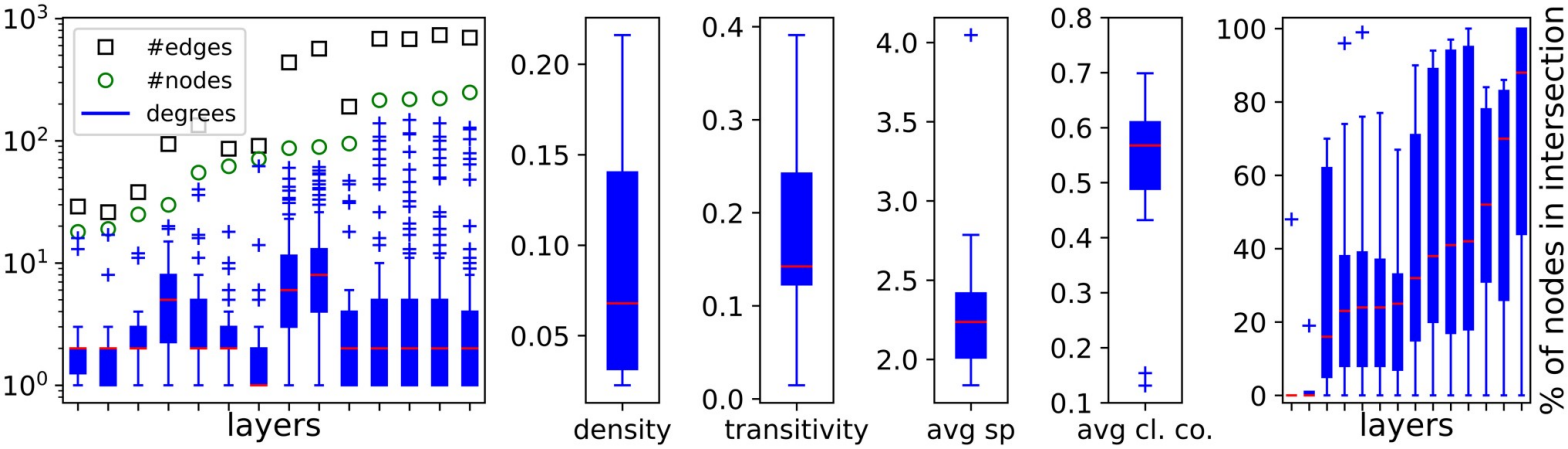
Statistics on layers in the USATN.

**Fig 2:**
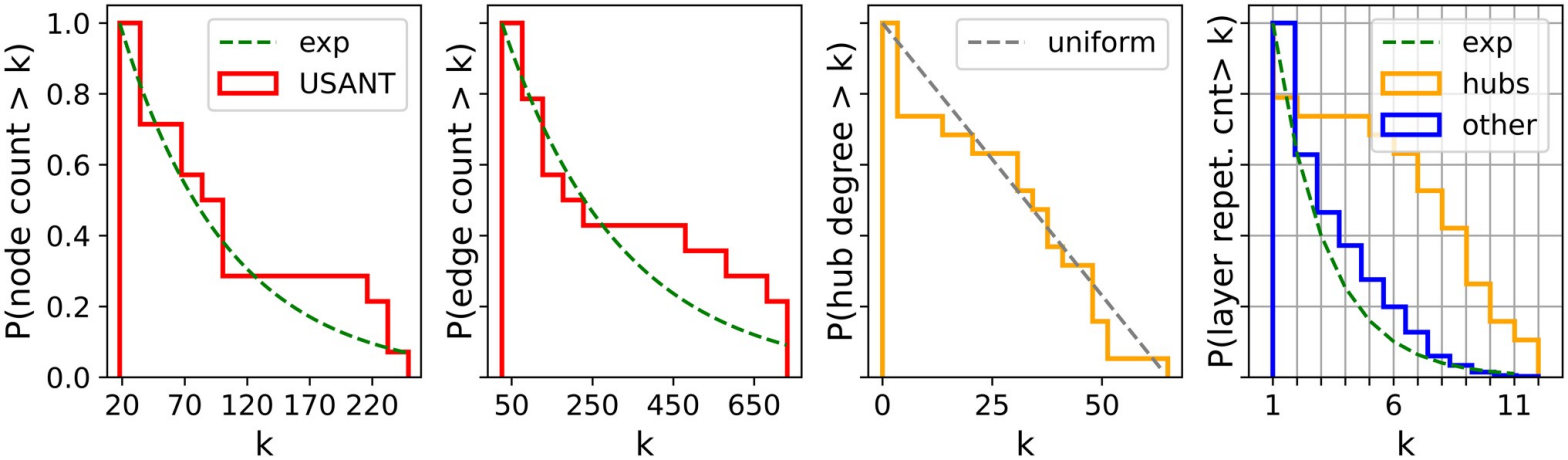
Statistics on layers in the USATN.

The second reference ATN we consider is composed of the US domestic airline connections retrieved from https://openflights.org in 2018. This airline network, hereinafter referred to as the USATN, consists of 14 layers, 436 nodes, and 4483 edges. Table 2 summarizes the main structural characteristics of this network, for each layer and the multiplex in the bottom row. Figs 1 and 2 provide further details, which are described below.

**Table 2:** Statistics on the layers and the multiplex (M) of the USATN.

layer id	node count	edge count	density	transitivity	avg degree	std degree	avg short. path	std short. path	avg clust. coeff.	std clust. coeff.
1	18	29	0.19	0.18	3.22	4.18	1.84	0.60	0.63	0.48
2	19	26	0.15	0.14	2.74	3.8	1.91	0.61	0.47	0.49
3	25	38	0.13	0.26	3.04	2.65	2.45	0.93	0.62	0.43
4	30	94	0.22	0.39	6.27	5.27	1.94	0.69	0.63	0.33
5	55	134	0.09	0.15	4.87	7.34	2.09	0.58	0.54	0.45
6	62	86	0.04	0.19	2.77	2.62	4.05	1.67	0.43	0.45
7	71	91	0.04	0.01	2.56	7.37	2.22	0.68	0.13	0.31
8	87	437	0.12	0.3	10.05	11.36	2.10	0.62	0.56	0.32
9	89	567	0.14	0.35	12.74	13.79	1.98	0.56	0.70	0.26
10	95	190	0.04	0.03	4.00	7.71	2.79	1.06	0.15	0.32
11	215	683	0.03	0.13	6.35	16.43	2.28	0.54	0.59	0.48
12	219	678	0.03	0.12	6.19	16.51	2.25	0.52	0.58	0.47
13	222	732	0.03	0.14	6.59	16.35	2.32	0.57	0.58	0.47
14	249	698	0.02	0.12	5.61	15.36	2.48	0.71	0.56	0.48
*min*	18	26	0.02	0.01	2.56	2.62	1.84	0.52	0.13	0.26
*max*	249	732	0.22	0.39	12.74	16.51	4.05	1.67	0.70	0.49
*μ*	104	320.21	0.09	0.18	5.50	9.34	2.34	0.74	0.51	0.41
*σ*	84.44	291.9	0.07	0.11	2.96	5.44	0.56	0.31	0.17	0.08
*M*	436	4483	0.05	0.32 (*)	20.56	46.28	3.28	1.45	0.56	0.41

(*) Values calculated with discarded multiple edges

The leftmost plot in Fig 1 shows the number of nodes and edges as well as a boxplot with node degrees for each layer. The ordering on the *x*-axis corresponds to layers sorted by the node count. The next four boxplots compile the values for the density, the transitivity, the average shortest path length, and the average clustering coefficient, respectively, collected for all layers in Table 2. The rightmost plot captures the overlap across all layers, where each boxplot corresponds to one layer, say *L*, and consists of the values

$$\begin{array}{c} p_{L^{\prime }}=\frac{|V_{L}\cap V_{L^{\prime }}|}{|V_{L}|}\cdot 100\% \end{array}$$ (1)

that are computed for every other layer in the network, *L*′ ≠ *L*, where *V*_*L*_ and *V*_*L*′_ denote the set of nodes in layer *L* and *L*′, respectively. The ordering of the layers, i.e., on the *x*-axis, is determined by the median of the boxplots.

The two plots from the left in Fig 2 display the cumulative histograms of the number of nodes and edges, previously listed in Table 2. The green curves represent the fittings to the exponential distributions that are used in the input for the ANGEL method (cf. Algorithm 2). Their parameters are listed in Table 3 alongside the KS-test statistics.

**Table 3:** USATN’s reference dependent parameters for the ANGEL model.

		*P*_*nodeL*_	*P*_*edgeL*_	*P*_*layerN*_
${\text{PDF}}_{e}\left(x,l,s\right)=s^{-1}e^{\left(-\frac{x-l}{s}\right)}$		PDF_*e*_ (*x*, 18, 86)	PDF_*e*_ (*x*, 26, 294)	PDF_*e*_ (*x*, 1, 2.19)
*KS-Test*	(*D*, *p*-value)	(0.395, 0.017)	(0.456, 0.003)	(0.369, 0.057)
	(***D***^+^, ***p*-value**)	(**0.177, 0.372**)	(**0.206, 0.268**)	(**0.247, 0.196**)
	(*D*^−^, *p*-value)	(0.395, 0.008)	(0.456, 0.002)	(0.369, 0.028)

The remaining two plots to the right in Fig 2 concern the statistics on *hubs*. Nodes of this kind form the core of network structures that are typical for flight connections. The affinity of a graph *G* (with edge-set *E*) to build hub-spoke formations can be measured using the *s-metric* value, $s_{m}^{G}=\sum_{(u,v)\in E}deg\left(u\right)\cdot deg\left(v\right)$ [50]. In [45], the s-metric formula is applied to set up an empirical definition of a hub. According to it, a node is identified as a hub if

$$\begin{array}{c} s_{m}^{L}\left(v\right)≔\frac{\sum_{u\in V_{L}\backslash \left\{v\right\}}deg\left(u\right)deg\left(v\right)}{|E_{L}{|}^{2}}>0.3, \end{array}$$ (2)

where *V*_*L*_ and *E*_*L*_ denote the set of nodes and edges in *L*, respectively.

Next, the degree distribution of hub nodes within the sub-network of the USATN they induce is shown in the third diagram from the left in Fig 2. This substructure plays a special role in the ANGEL model [45] (cf. Algorithm 2, step 7) and is created after nodes and before edges are assigned to layers. The configuration model applied in that context is based on the assumption that degrees of nodes in this sub-network follow a uniform distribution. According to a KS-Test, this degree distribution fits the uniform distribution on the interval [0, 65] (shown in the plot) with the *p*-value of 0.6139 for the maximum negative deviation *D*^−^ = 1.105.

Finally, the rightmost plot in Fig 2 refers to the term *layer repetition count per node* in Algorithm 2 and displays the histograms on how many layers a node shares. The orange line corresponds to the layer repetition count per hub, whereas the blue one to non-hubs. The fitting curve to the latter distribution, *P*_*layerN*_, is applied in the first step of the ANGEL model (cf. Algorithm 2, step 4), where nodes are allocated to the layers. We refer to Table 3 for the parameters and KS-test statistics of this exponential probability function.

## Topological analysis

This section is divided into two subsections, each presenting the performance of the synthetic models under study—BINBALL, STARGEN, and ANGEL—against the reference networks, the EATN and the USATN, respectively. In addition, each subsection consists of two parts: the first takes into account the entire multiplex, the second focuses on the layers.

To begin with, let us provide some details of the evaluation methodology adopted. The three algorithms for the generation of synthetic networks are initialized with equal input data in terms of number of nodes, edges, and layers that come from the respective real network, as displayed in Table 4, alongside the additional input for the ANGEL algorithm. The remaining parameters are fixed as specified in [6, 44, 45].

**Table 4:** Input parameters for the generative algorithms.

				EATN	USATN
ANGEL	STARGEN	BINBALL	*n*	417	436
			*m*	3588	4483
			*l*	37	14
		*P*_*edgeL*_		PDF_*e*_ (*x*, 33.99, 62.86)	PDF_*e*_ (*x*, 26, 294)
	*P*_*layerN*_			PDF_*e*_ (*x*, 1, 3.88)	PDF_*e*_ (*x*, 1, 2.19)
	*P*_*nodeL*_			PDF_*e*_ (*x*, 34.99, 19.75)	PDF_*e*_ (*x*, 18, 86)
	*p*			10%	10%

PDF_*e*_ (*x*, *l*, *s*) as defined in Table 3

**Fig 3:**
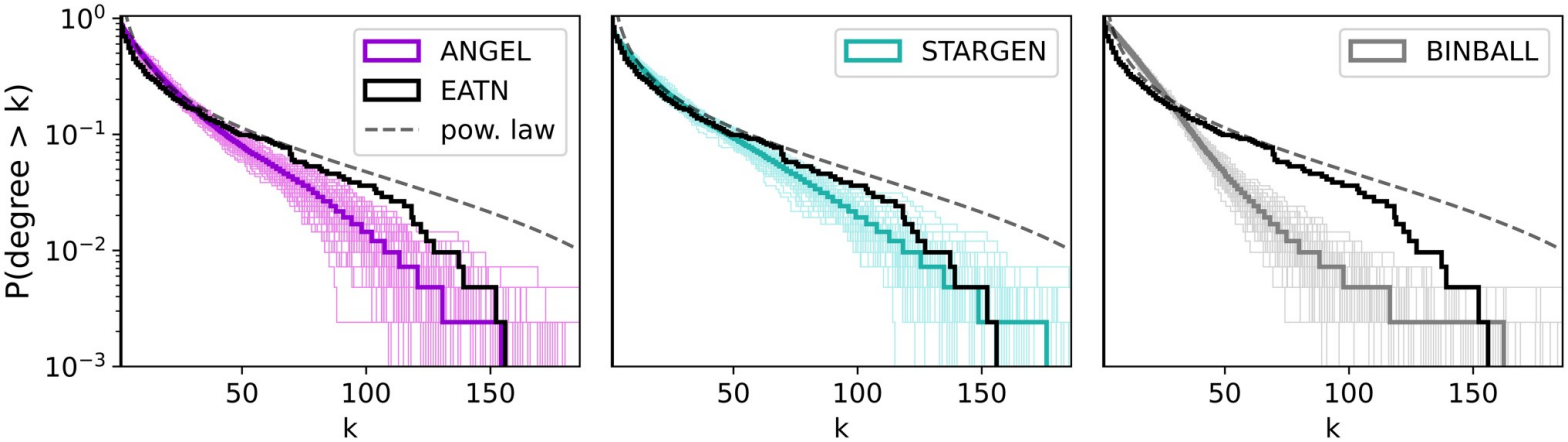
Multiplex degree distribution.

The statistics we present base on 100 multiplex replicas generated for each synthetic model or one multiplex randomly selected from the sample. Each multiplex is represented by the set of layers it is composed of, and viewed as a multigraph. When an average line over the 100 samples is plotted, it is calculated in the following way: for each sample, *y*-values are sorted, then per *x*-value the average over 100 *y*-values is calculated. Usually, a thick colored line is the average and is drawn over the group of faded lines that correspond to 100 replicas in the background. The color code for the models and the reference is introduced in Fig 3 and carried throughout the section.

### Validation of the synthetic models versus the EATN

#### Topological comparison of the multiplex networks

A macroscopic view of the synthetic multiplexes compared to the EATN is provided by the values compiled in Table 5. It displays, line by line, the minimum, average, and maximum values for the respective network statistics. The columns, except for the reference, relate to the minimum, mean, and maximum values per row, calculated over the 100 synthetic replicas with respect to the model given in the heading. As one can observe, the values of the synthetic networks differ from the EATN within a small range and especially the average values over the replicas stay close to the reference.

**Table 5:** Network statistics. 100 multiplex replicas per model versus the EATN.

		EATN	ANGEL			STARGEN			BINBALL		
			min	mean	max	min	mean	max	min	mean	max
*degree*	*mean*	17	16	17	17	16	17	17	16	17	17
	*max*	156	109	154	246	109	165	253	89	164	289
*closeness centrality per node*	*min*	0.20	0.23	0.27	0.30	0.19	0.25	0.29	0.00	0.24	0.28
	*mean*	0.37	0.39	0.40	0.41	0.39	0.39	0.41	0.39	0.40	0.41
	*max*	0.55	0.53	0.56	0.61	0.53	0.56	0.61	0.51	0.56	0.64
*average short. path per node*	*min*	1.82	1.64	1.78	1.88	1.64	1.77	1.89	0.5	1.74	1.94
	*mean*	2.74	2.48	2.56	2.62	2.48	2.59	2.65	2.48	2.53	2.58
	*max*	4.86	3.38	3.72	4.37	3.43	4.00	5.13	3.56	4.03	4.95
*density*	*multigraph* (*)	417	407	416	417	417	417	417	417	417	418
	*simple* (***)	343	361	375	384	355	370	385	382	391	398
*transitivity* (**)		0.30	0.19	0.21	0.25	0.18	0.21	0.24	0.11	0.12	0.14
*average path length*		2.75	2.48	2.56	2.62	2.48	2.58	2.65	2.48	2.53	2.58
*betweenness centrality* (*)		42	36	38	39	36	38	40	36	37	38

(*) value ×10^−4^,

(**) values calculated with discarded multiple edges,

(***) both

Next, we turn to a more detailed analysis of the generated multiplexes. In the first statistic we consider, the degree histograms shown in Fig 3, it can be noted the comparably good performance of ANGEL and STARGEN networks and the trailing off effect by the BINBALL model. This trend will continue.

**Fig 4:**
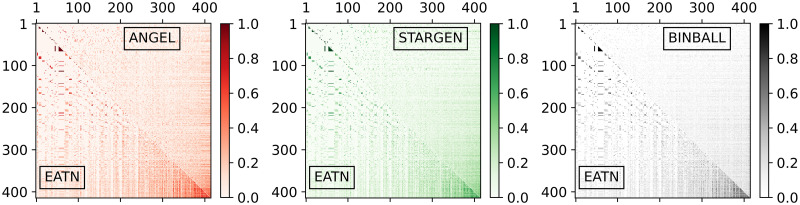
Multiplex cosine centrality.

The plots in Fig 4 give an insight into structural similarity aspects of the networks. Per plot, one instance from the sample of the considered model is randomly selected and compared with the reference. The darker the shading is, the higher the cosine similarity per node-pair is (*i.e*., the number of common neighbors of the two nodes normalized by the geometric mean of their degrees). Nodes on both axes are sorted by degree. The darkest zone in the lower right corner of the EATN triangle indicates that high degree nodes, *i.e*., hubs, are strongly connected among themselves. Apparently, this tendency is less pronounced for synthetic networks. However, ANGEL’s relatively diverse transition stands out against the smoother textures of STARGEN and BINBALL.

**Fig 5:**
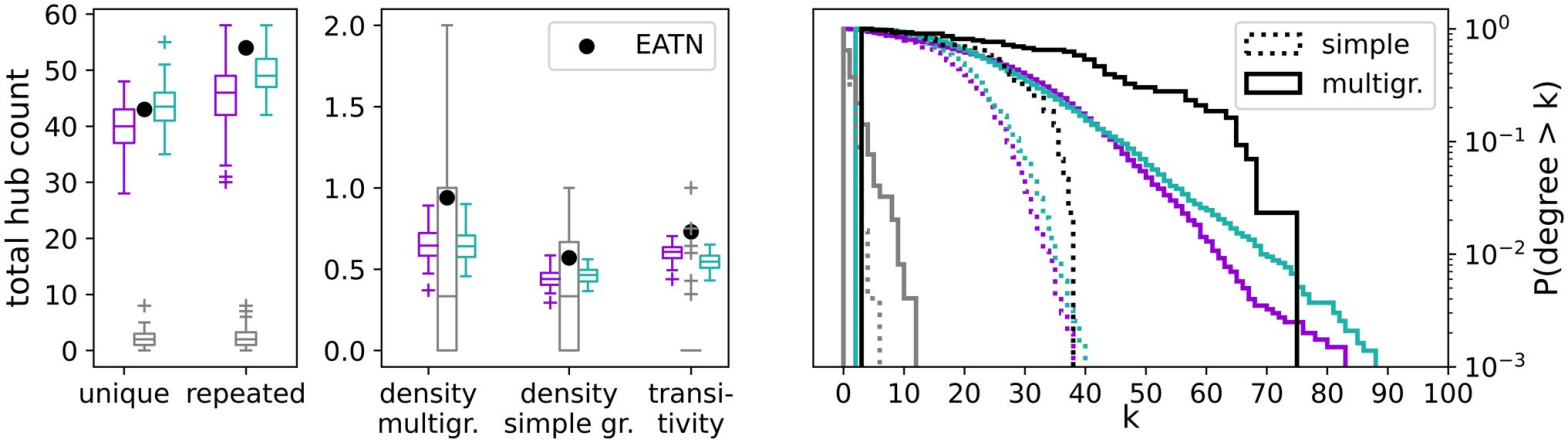
Statistics on the sub-network induced by the hubs.

In Fig 5, we present statistics on the sub-network of the multiplex induced by the hub nodes, cf. Eq (2). About 20% of the hubs in the reference network share their central role across the layers. ANGEL and STARGEN networks build hubs in number close to the EATN, but with fewer repetitions, as it can be read from the first plot on the left. Since airlines naturally offer connections between the central airports, the hub-subnetwork is a multigraph. According to the next chart in the row, every connection is offered twice on average in the EATN. When the repeated edges are discarded from the multigraph, the density is reduced by about a half. The same applies to ANGEL and STARGEN.

**Fig 6:**
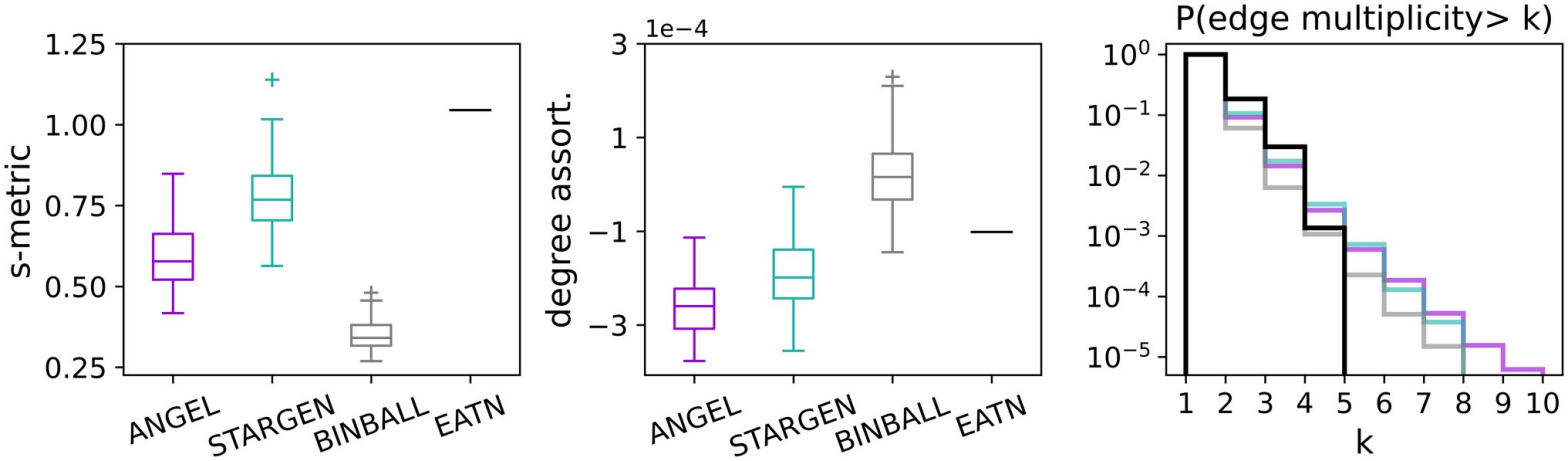
S-metric, degree assortativity, and edge multiplicities of the multiplex.

The high transitivity values confirm that the hubs are very well connected both in the EATN as in ANGEL and STARGEN networks. However, hubs in these replicas achieve higher maximum degrees than the reference, as the average degree distributions of the hub-subnetworks in the right plot show. Both histograms, for the ANGEL and STARGEN model, approach the uniform distribution. In ANGEL, it results from the application of the configuration model on uniformly distributed degrees in the creation of the hub sub-network (cf. Algorithm 2, step 7). In STARGEN, the degree distribution is definitely a consequence of the preferential attachment method. Considering all charts in Fig 5, BINBALL replicas feature very poorly hub-spoke formations. Only a few hubs are counted, which leads to small graphs they span and deviations in the statistics. The weakness of BINBALL in generating networks spanned on hubs is confirmed by the low s-metric value (cf. Section *Background*), as shown in the bottom left plot in Fig 6. The boxplots show the s-metric values computed for the entire multiplex and normalized with the squared number of edges. Notice that the latter quantity is the same for each model or close to that in the reference. Both ANGEL and, in particular, STARGEN multiplexes consist of well-exposed hub-spoke formations, but not as strong as the EATN.

The middle chart of Fig 6 confirms the presence of hubs in all the considered networks. The degree assortativity is negative and close to 0 for ANGEL and STARGEN as in the EATN. Such values are expected in networks with hub-spoke structures, where low degree nodes are satellites of the strongly connected centers. Here again, we observe that hubs in BINBALL have the weakest attraction.

Finally, in the right plot in Fig 6, we show the average distributions of edge repetitions in the multiplex, *i.e*., how often one connection is offered by several airlines. As it can be noted, while the majority of the edges in all networks including the reference are simple, the replicas have fewer repeated edges, but with higher frequencies, compared to the EATN.

#### Topological comparison of the layers

**Fig 7:**
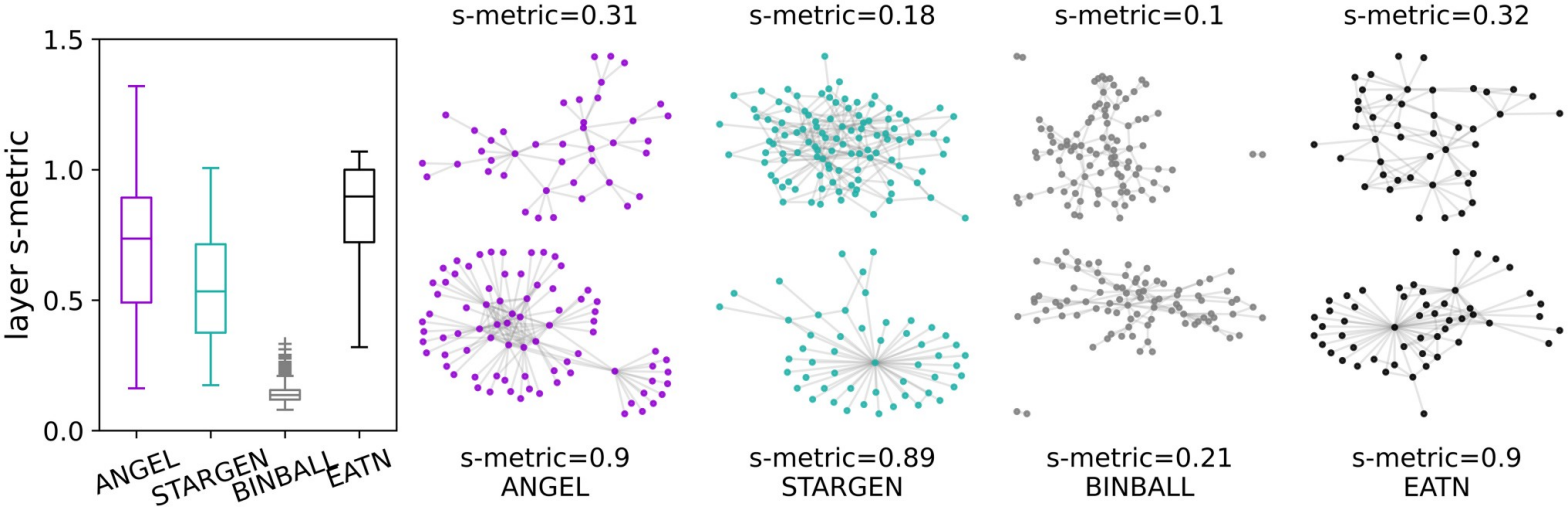
S-metric and layer formations.

Breaking down the s-metric values on the layer level, we observe from the left chart in Fig 7 that the values for the EATN and STARGEN decrease and for ANGEL increase compared to the global multiplex value (cf. Fig 6 left plot). The boxplots aggregate s-metrics calculated for every layer in the 100 replicas per each model or for the 37 layers in the case of the EATN. The values are normalized with the squared number of edges in the respective layer. The remaining plots in Fig 7 shall give an impression of which layer formation corresponds to low (top row) and high (bottom row) s-metric values. Each pair of layers per model belongs to one multiplex randomly selected from the 100 samples. In addition, the ANGEL and STARGEN examples have been chosen so that their s-metric value is close to that of given at the reference. In the case of BINBALL, we show layers with the minimum and maximum s-metric value because they do not fulfill the above restriction. The displayed structures confirm the weak hub-spoke formations generated by the latter model.

**Fig 8:**
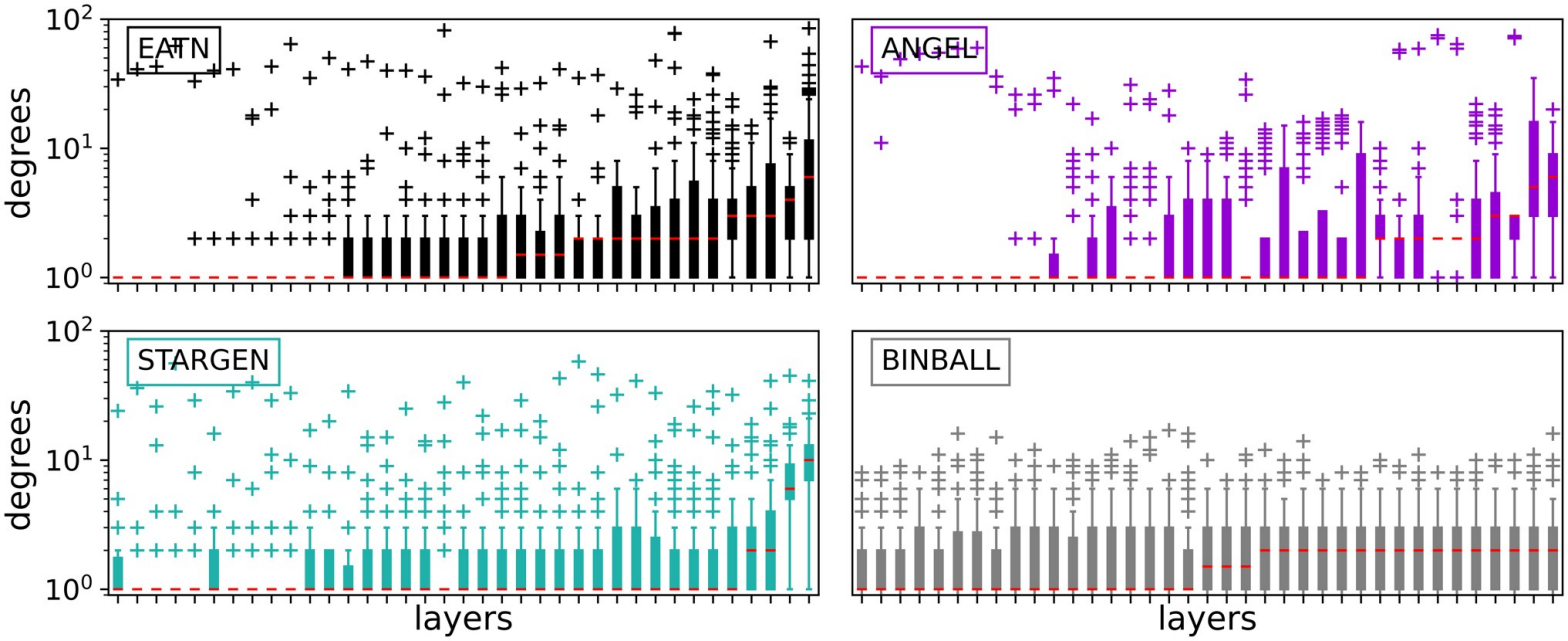
Node degrees within the layers.

Further statistics on the topological structure of the layers, viewed as a coherent mixture composing a multiplex, are shown in Figs 8 and 11. The first group of charts, Fig 8, offers a consolidated view on the degree distribution within the individual layers of a multiplex, the reference or a random selection from the 100 replicas.

Per chart, each boxplot shows all node degrees within one of the 37 layers of the multiplex. The ordering of the boxplots is performed with respect to the median followed by the mean value. Compared to the other models, ANGEL layers show the most diversity in the degrees within the layers, even if they have more leaves or high degree nodes than the reference. The STARGEN algorithm creates homogeneous layer formations where the majority of nodes have a degree less than 2. The uniform structure of BINBALL layers is striking again.

**Fig 9:**
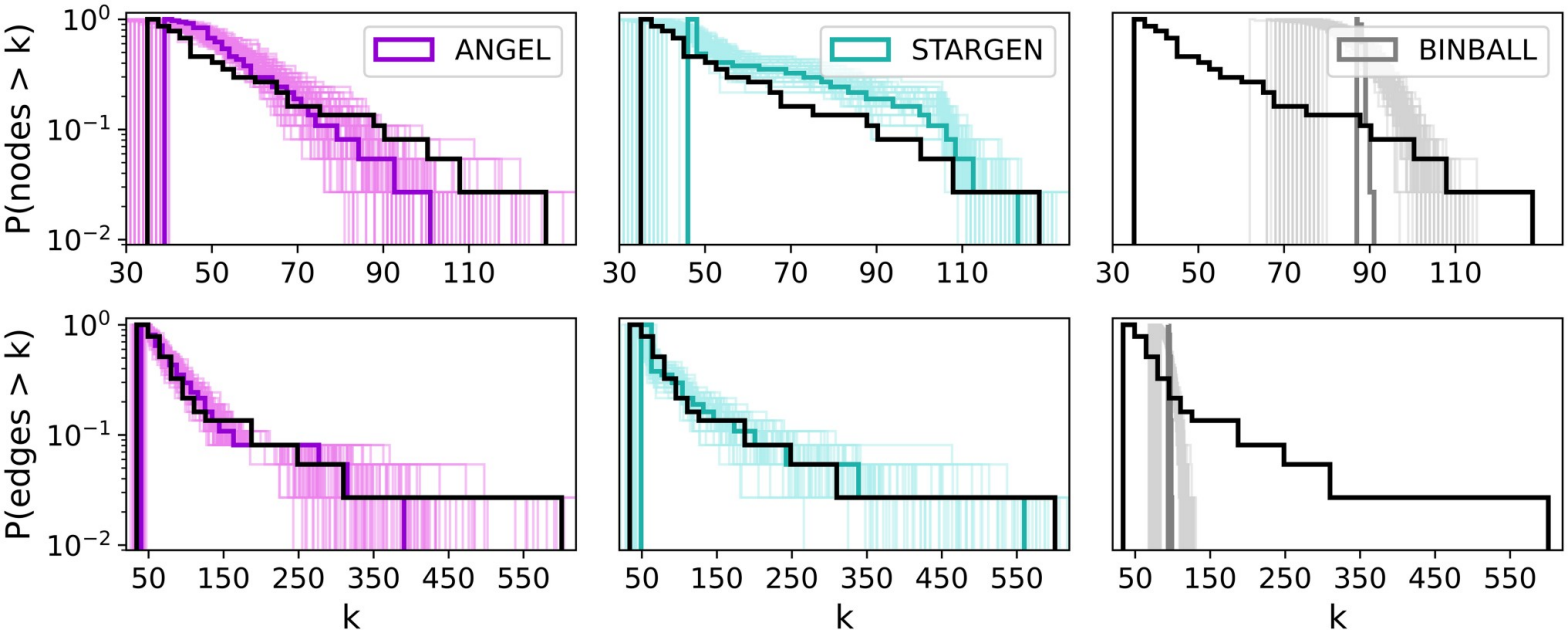
Layer node and edge number distributions.

The next group of charts, Fig 9, presents histograms on node and edge numbers over a layer set per multiplex in the 100 sample. The thick line is the average distribution, the faded ones correspond to every single multiplex in the 100 sample. Although the fit to the reference for the node and edge number, *P*_*nodeL*_ and *P*_*edgeL*_, is used in the input for ANGEL, this model struggles to mimic the outlier of the EATN, the layer with 128 nodes and 601 edges. The replication of layers with a smaller number of nodes and edges is more accurate. STARGEN offers the best performance in terms of mimicking the edge size, but mostly produces layers with higher node numbers than the reference, except for the largest. The BINBALL distributions are not surprising when recalling the uniform layer size constraints in the input.

**Fig 10:**
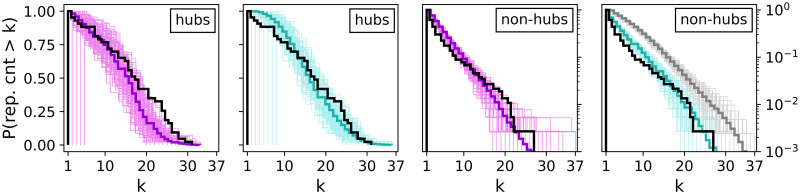
Layer repetition counts per node.

The histograms in Fig 10 merge layer repetition counts per node. The value indicates how many layers a node belongs to. The nodes are divided into hubs (left plots) and non-hubs (right plots). Due to the insufficient number of hubs, the BINBALL curves are not plotted for hubs and hence the non-hub curve relates to almost all nodes. Considering hub repetitions in layers, we observe that STARGEN networks usually have hubs present in almost all layers, not like the EATN or ANGEL, and in ANGEL, fewer hubs with a higher repetition frequency than in the reference can be observed. Both ANGEL and STARGEN show overall a very good performance with respect to non-hub repetition counts. Recall that the fit to the reference curve for non-hubs, *P*_*layerN*_, is used in the input for ANGEL.

**Fig 11:**
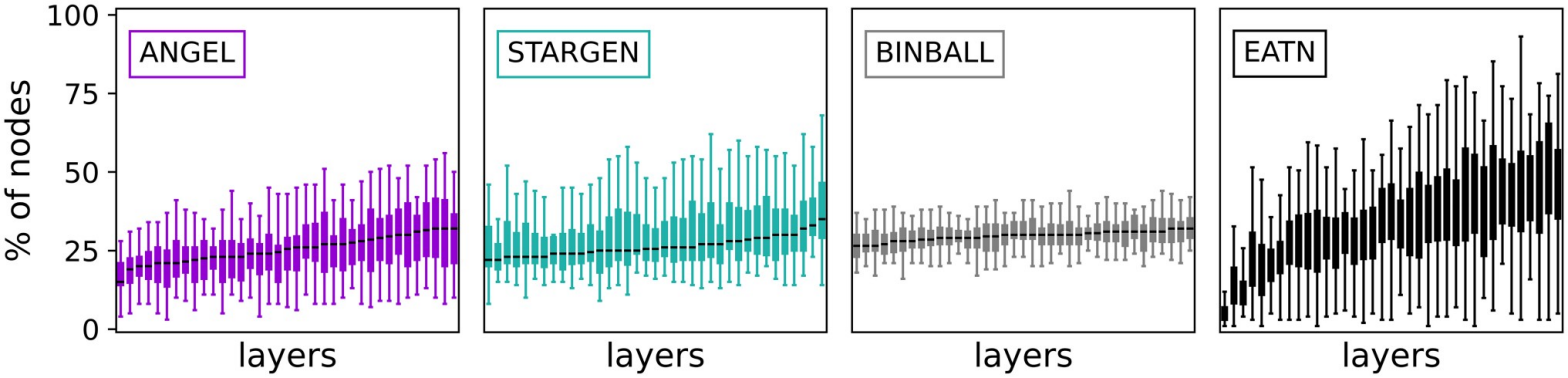
Percentage of nodes in layer intersection.

On the contrary, the structural characteristic of the EATN we consider next is difficult to replicate. The charts in Fig 11 offer another view on the layer node-set overlap. The boxplots correspond to the statistic defined in Eq (1), and express the percentage of nodes each layer shares with the others. For every chart except the rightmost one showing the reference network, a multiplex is randomly selected from the 100 replicas of the respective model. Compared to the reference, STARGEN and BINBALL form a quite uniform pattern. ANGEL shows a growing tendency, but only a few outliers reach the overlap over 50%, the majority of the EATN layers is around or close to.

### Validation of the synthetic models versus the USATN

#### Topological comparison of the multiplex networks

At a first glance, the standard network statistics shown in Table 6 reveal that the USATN multiplex is more difficult to replicate by the synthetic models than the EATN. Remarkably, ANGEL, STARGEN, and BINBALL appear to be on average comparable to one another.

**Table 6:** Network statistics. 100 multiplex replicas per model versus the USATN.

		USATN	ANGEL			STARGEN			BINBALL		
			min	mean	max	min	mean	max	min	mean	max
*degree*	*mean*	20	20	20	20	20	20	20	20	20	20
	*max*	352	119	189	282	119	195	305	105	191	426
*closeness centrality per node*	*min*	0.13	0.24	0.28	0.33	0.21	0.26	0.30	0.00	0.25	0.30
	*mean*	0.32	0.40	0.42	0.43	0.40	0.41	0.43	0.41	0.42	0.42
	*max*	0.51	0.54	0.59	0.70	0.55	0.58	0.63	0.53	0.58	0.68
*average short. path per node*	*min*	1.96	1.43	1.68	1.86	1.58	1.71	1.82	0.50	1.70	1.87
	*mean*	3.27	2.34	2.43	2.56	2.38	2.46	2.55	2.40	2.44	2.47
	*max*	7.25	3.05	3.52	4.23	3.38	3.91	4.69	3.30	3.86	5.47
*density*	*multigraph* (*)	473	463	473	473	473	473	473	473	473	473
	*simple* (***)	275	428	445	456	421	429	439	430	441	449
*transitivity* (*)		0.32	0.17	0.21	0.26	0.18	0.20	0.22	0.12	0.13	0.15
*average path length*		3.27	2.34	2.43	2.56	2.38	2.46	2.55	2.40	2.44	2.47
*betweenness centrality* (*)		52	31	33	36	32	34	36	32	33	34

(*) value ×10^−4^,

(**) values calculated with discarded multiple edges,

(***) both

**Fig 12:**
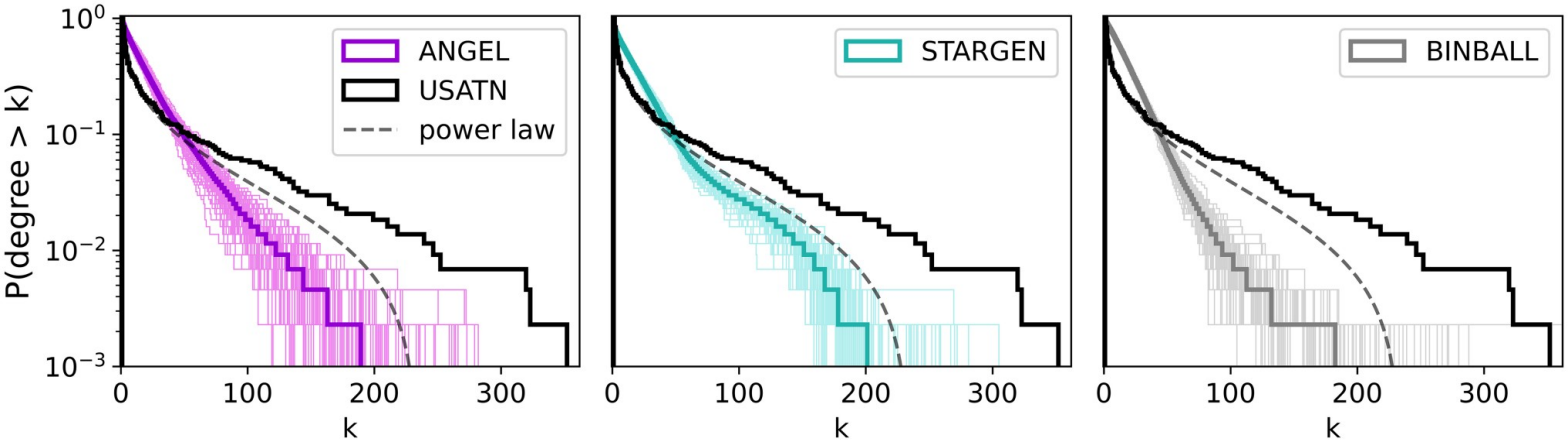
Multiplex degree distribution.

**Fig 13:**
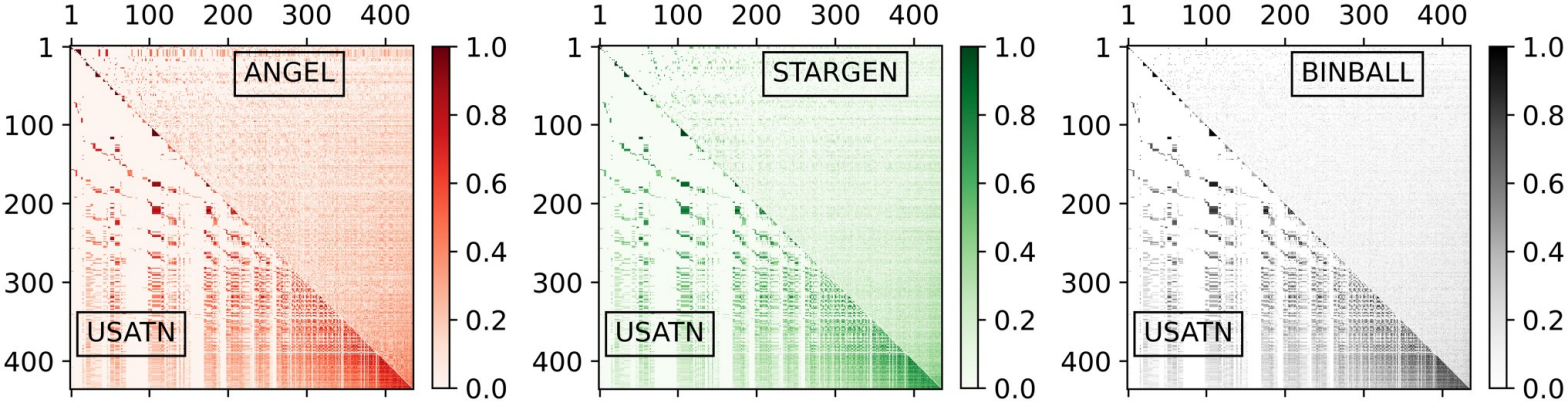
Multiplex cosine similarity.

**Fig 14:**
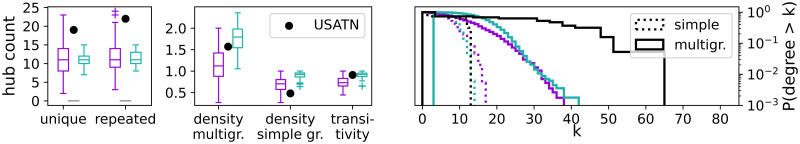
Statistics on the sub-network induced by the hubs.

**Fig 15:**
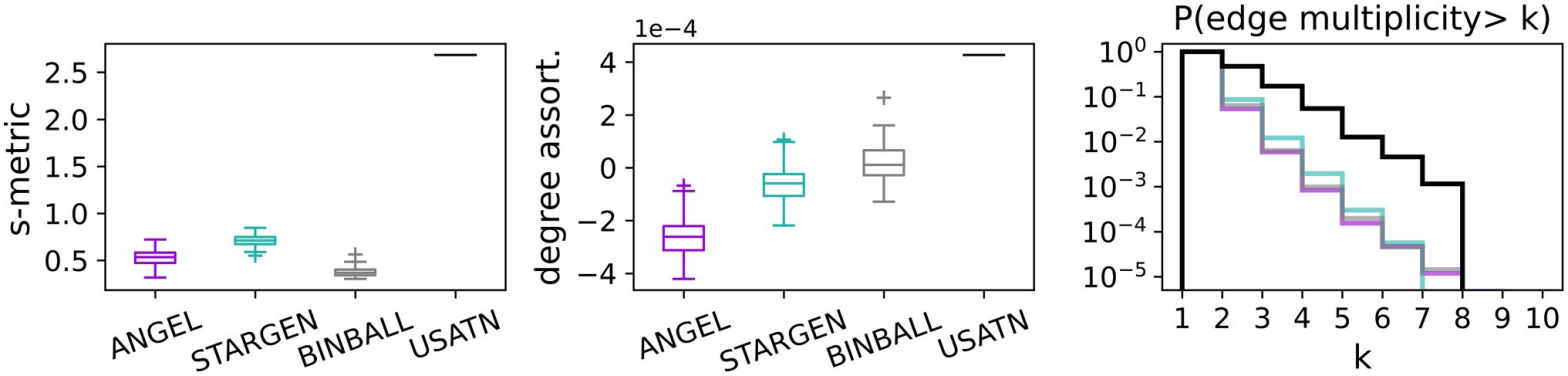
S-metric, degree assortativity, and edge multiplicities of the multiplex.

Considering the statistics plotted in Figs 12–15, ANGEL and STARGEN compete with each other as they have done in the validation against the EATN. Both fail to approximate the degree distribution of the USATN, however, follow its power-law fitting (Fig 12).

In Fig 13, the pattern created by the USATN in the charts for comparing the cosine similarity indicates that the overlap of the neighbors is greater, the higher the degree of the node. Recall that nodes are sorted by the increasing degree on both axes. This tendency can be observed in the synthetic models, and especially in ANGEL, but not to the extent as in the reference.

Plots in Fig 14 concern the graph induced by the hubs that have been previously identified in the layers. As we have learned from the validation against the EATN, nodes in BINBALL fail to be tagged as hubs. In ANGEL, only outliers reach the count of the reference, and it may happen that very few hubs are present in the multiplex. The hub numbers in STARGEN networks stay steadily around half of the USATN. The small repetition rate of hubs in this reference network is difficult to replicate in the synthetic multiplexes. As the values of the densities and the degree distribution of the networks with discarded multiple edges (labeled as a simple graph in the middle and right plot) indicate, the hub sub-networks in ANGEL and STARGEN form connected structures comparable with the reference. However, from the deviations between the curve, we close on fewer repetitions of the edges in the synthetic multigraphs.

It is not surprising that the s-metric values of the multiplexes, presented in the left plot in Fig 15, are as high as when replicating the EATN. All synthetic models are designed to mimic airline networks with a predetermined affinity to form hub-spoke structures. However, they fail to reproduce the extremely high s-metric value of the USATN. The degree assortativity values of the replicas are also similar to those from the EATN, cf. the middle plot in the row. Clearly, the positive value for the USATN correlates to the cosine similarity charts, where congestion around high degree nodes has been observed.

The plot to the right in Fig 15 confirms the presence of edges with a high multiplicity in the USATN, which has already been observed when analyzing the hub sub-network. The generative models, especially ANGEL, manage to produce multiplexes having edges with a comparably high repetition rate as the reference, but with a lower frequency.

#### Topological comparison of the layers

Following the outline of the analogous section when validating against the EATN, we look first at the layer structures created by the generative models.

**Fig 16:**
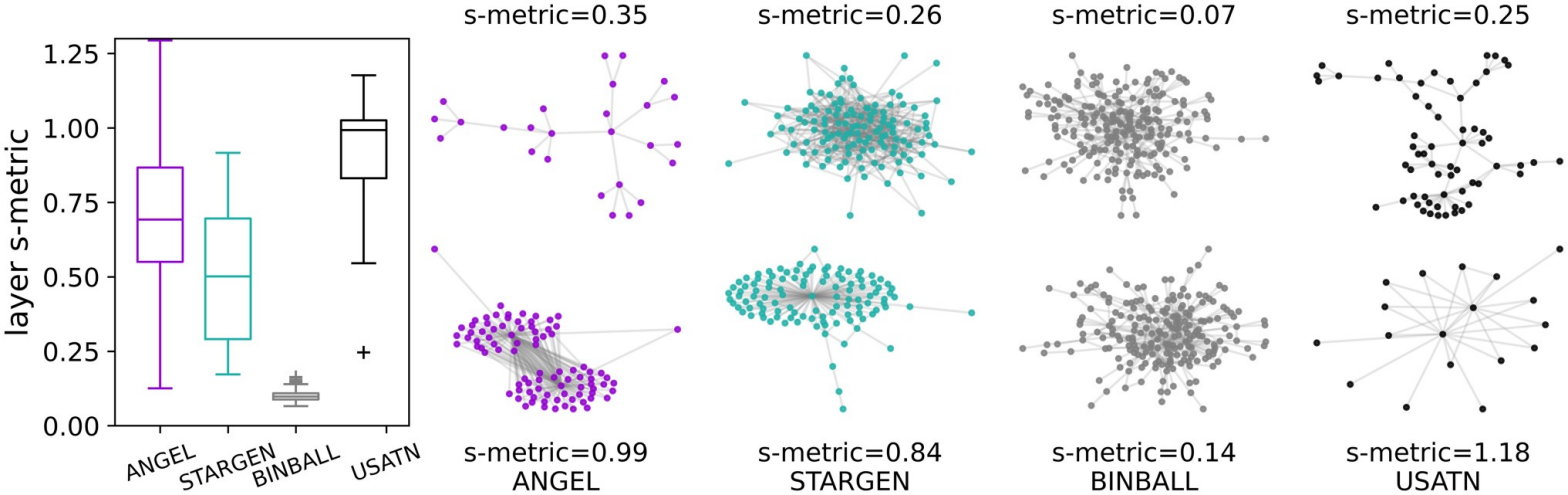
S-metric and layer formations.

In Fig 16, alongside the boxplots consolidating the s-metric in every layer within the 100 replicated multiplexes or in the 14 of the USATN, a layer having a minimum (top) and maximum (bottom) s-metric value is displayed for one synthetic multiplex example per model; the same applies for the reference.

The following statistics presented in Figs 17–20 give an overview of the structural properties of layers that form synthetic multiplexes, analogous to Figs 8–11, this time compared to the USATN.

**Fig 17:**
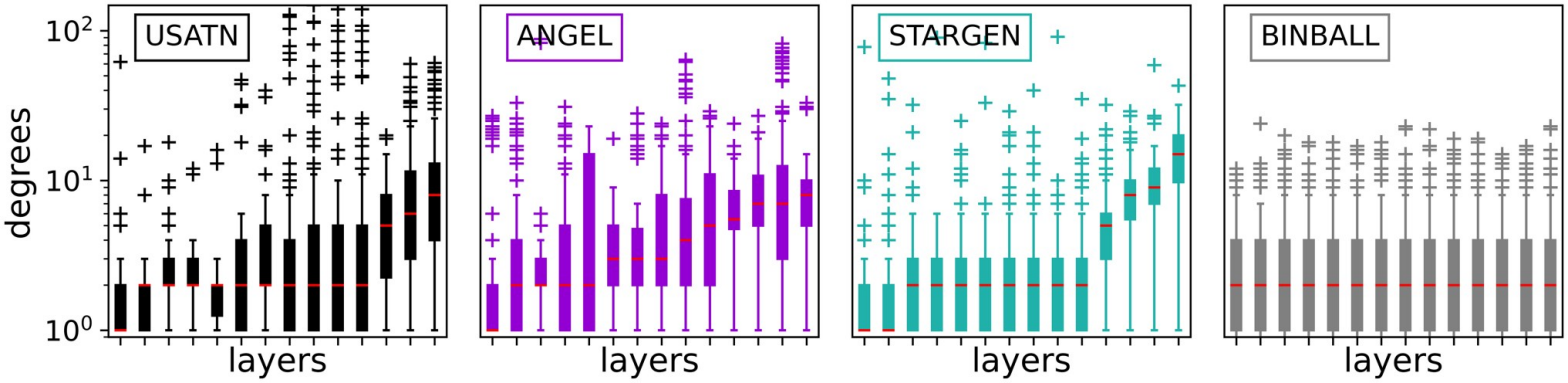
Node degrees within the layers.

Considering the boxplots with node degrees in a set of layers of one randomly selected multiplex from the 100 sample (Fig 17), we observe that although ANGEL layers have more leaves compared to the reference, they have nodes in a wider range of degrees. This diversity is missing in STARGEN. Here, either low or high degree nodes have the majority within a layer.

**Fig 18:**
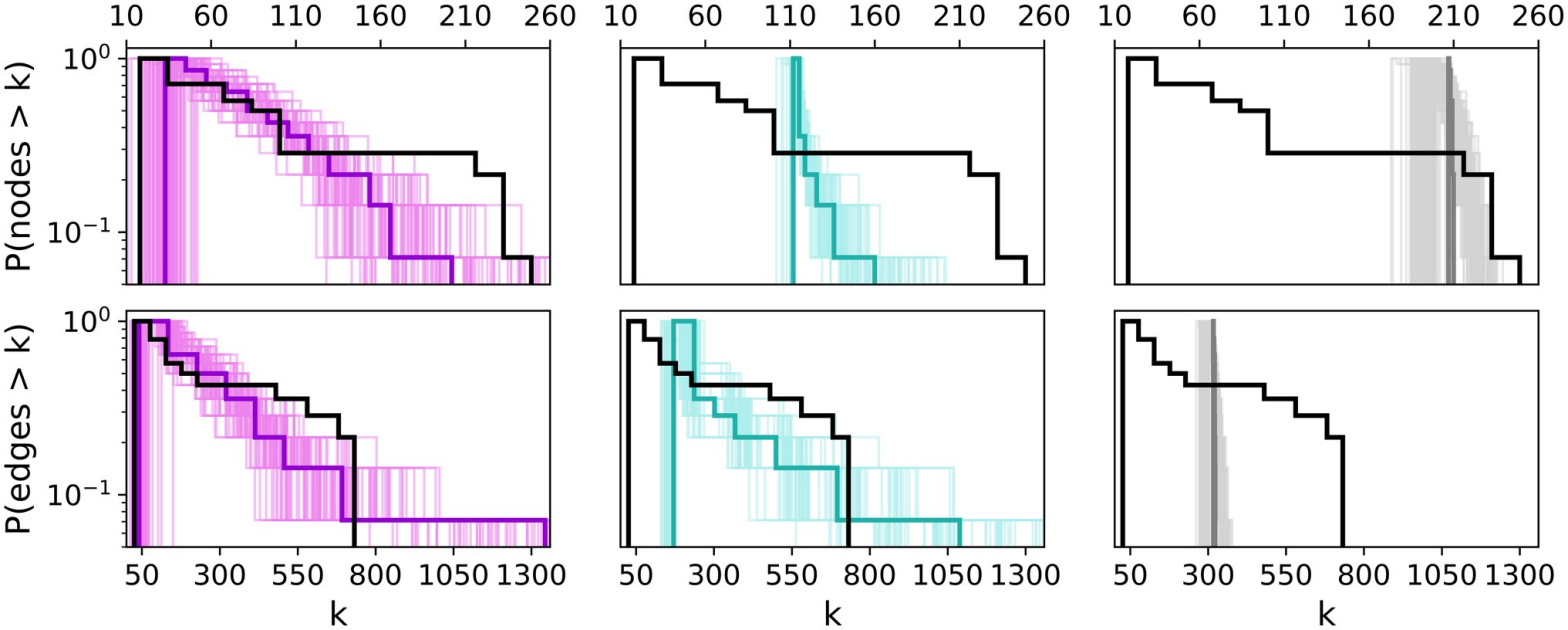
Layer node and edge number distributions.

The homogeneous trend in STARGEN is continued with regard to the number of nodes in the layers (Fig 18), which remains in a narrow range between 100 and 200. The performance is much better when layer edge numbers are replicated. However, this is to be expected, since the model is initialized with the fit to the reference distribution studied here. The same applies to ANGEL. In both cases, the exponential fitting to the layer edge number distribution, *P*_*egdeL*_, leads to remarkably long tails.

**Fig 19:**
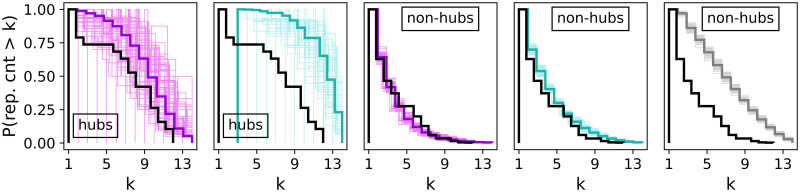
Layer repetition counts per node.

As it can be observed in Fig 19, the hub repetition count per layer is highly volatile, in ANGEL as well as in STARGEN. Recall that in BINBALL no hubs are counted. Nevertheless, the average curve for ANGEL progresses close to but above the reference. Considerably higher hub repetitions can be observed in STARGEN. On the contrary, the non-hub curves of these two models meet the reference.

**Fig 20:**
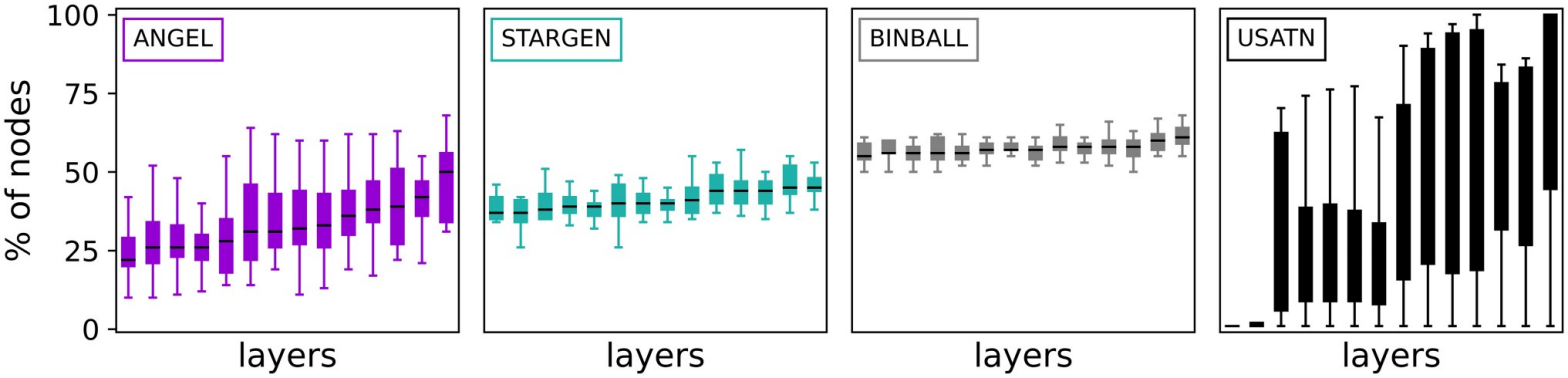
Percentage of nodes in layer intersection.

The statistic presented in Fig 20 proves a weakness of all generative models that already has been observed when validating the EATN. The proportion of nodes that a synthetic layer has in common with others remains in a small range or is almost the same as in case of STARGEN or BINBALL. Nevertheless, the ANGEL model is the best-performing method.

The performance of the BINBALL model on statistics concerning the layer structure (Figs 17–20) is comparable to the previous reference. Remarkably, the percentage of nodes in layer intersection is much higher than when replicating the EATN.

#### Individual layer replication using ANGEL

**Fig 21:**
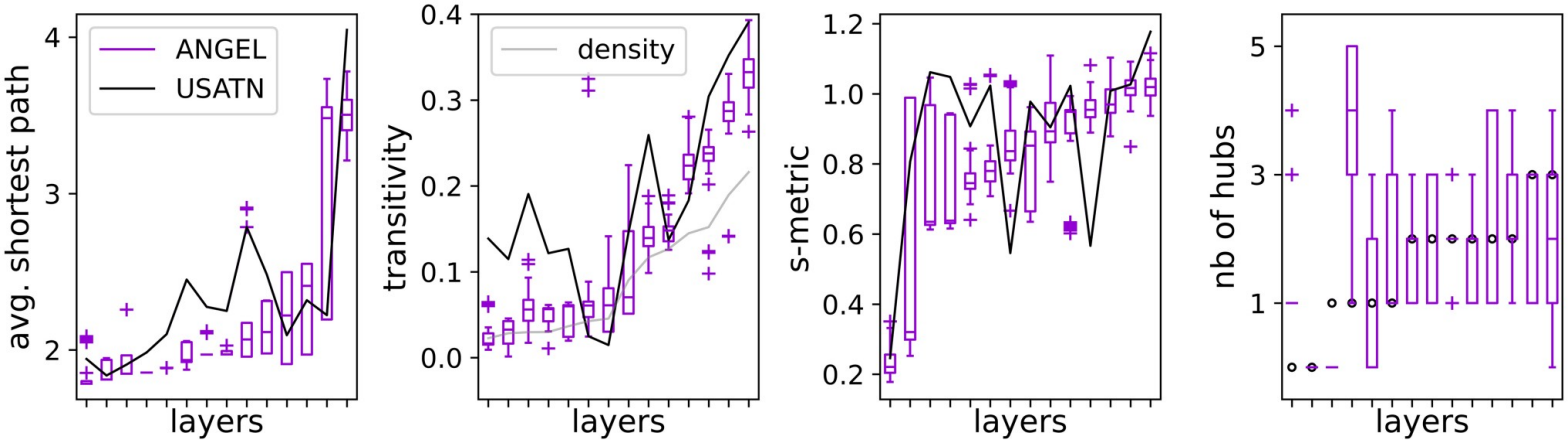
Statistics on USATN layer replication with ANGEL.

Layers in the STARGEN and BINBALL models grow simultaneously and cannot be distinguished as exact replicas of particular layers of the reference. Not as in the ANGEL model, where the multiplex is built layer by layer, and therefore it is possible to imitate individual layers of the replicated multilayer network. In Fig 21, we present a selection of statistics introduced in [45] for validation of ANGEL’s single-layer replication against the EATN, here computed with respect to the USATN.

From the left, the average path lengths, the transitivity, the s-metric, and the number of hubs (cf. Eq (2)) are displayed. In every plot, one boxplot collects the measured value calculated for 100 replicas of one USATN layer tagged on the *x*-axis. The black solid lines connect the values of the reference layers. In the second plot, the grey line relates to the density values, that result in the same for the synthetic and real layers. Despite a few larger discrepancies, the overall approximation of the metrics is noticeable.

## Resilience analysis

Having reviewed the structural properties of the synthetic networks we turn to test their resilience. Our first goal is to compare the resilience of the synthetic networks to the reference ones. Moreover, we want to investigate a way of building an optimally structured airline network with minimal failure effects; for this purpose, we will resort to the flexibility of the ANGEL model to balance between the number of hub-spoke and the point-to-point layers.

Like any other type of network, air traffic networks are rated according to their resilience. The closure of a site (i.e., an airport), or even a loss of a link (i.e., a connection between two airports) can have a huge impact on an air transportation network. The enormous costs, delays in hours or even days, and the entire logistical background that has to be diverted should not be underestimated. Keeping track of the succession of the failure of sites or bonds that leads to the total breakdown of the network is also a related aspect to investigate.

In our study, we simulate and analyze various attack strategies for site as well as bond percolation tasks. In general, in addition to a random attack (i.e., based on a random ordering of either the nodes or the edges), we contrast it with a structured sequence of failures, such as starting with nodes with the highest degree, or edges that connect them. We now elaborate on each of the proposed percolation strategies.

### Site percolation

We define a global strategy and a local strategy for site percolation. In the global approach, the nodes are detached according to their closeness centrality with respect to the multiplex, whereas the local strategy utilizes the degree centrality with respect to the layers as criterion. If a node belongs to several layers, the maximum degree value determines the order. In both cases, the global closeness and the local degree centrality, nodes are removed in either decreasing or increasing order from the multiplex and so its layers.

### Bond percolation

In the bond percolation, we pursue two main strategies in addition to the random one. In the first approach, the edges are sorted according to the degree of their end nodes. Either the minimum or the maximum end degree is taken for the ordering, i.e., for any two edges *e*_1_ = (*u*, *v*) and *e*_2_ = (*r*, *s*) we consider

(u,v)≺(r,s)⇔f(deg(u),deg(v))<f(deg(r),deg(s)),

where f here denotes a placeholder for function *min* or *max*.

In the case of multiple edges, the local degrees in the biggest layer with respect to the number of edges are considered. In the second strategy, the edges are selected for removal depending on their multiplicity. In every percolation scheme we pursue, edges are removed from the multiplex and layers one by one. If an edge is multiple, the connection in the multiplex is only broken after all multiplicities of the edge have been removed. Note that, since layers are simple graphs, the layer an edge belongs to is uniquely determined, therefore only one layer at a time is affected when a non-singular edge is detached.

### Validation against reference networks

**Fig 22:**
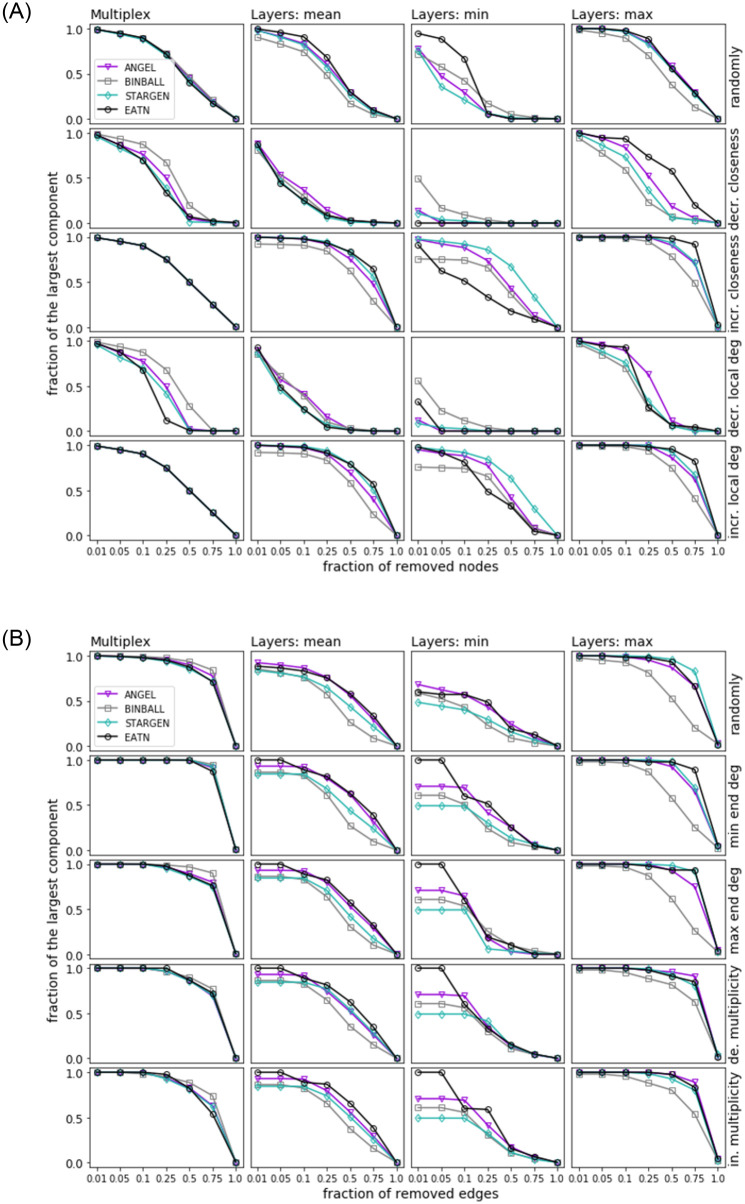
Site (top) and bond (bottom) percolation. Synthetic networks versus the EATN.

**Fig 23:**
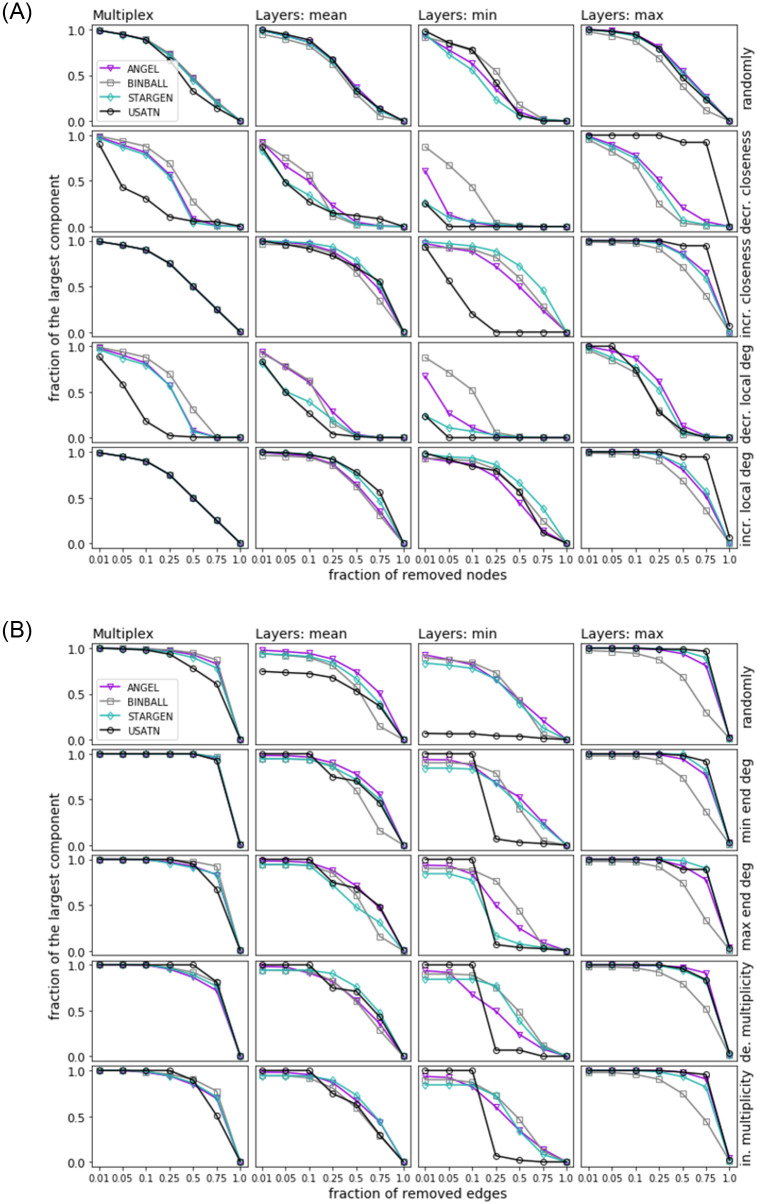
Site (top) and bond (bottom) percolation. Synthetic networks versus the USATN.

Figs 22 and 23 show the results of site and bond percolation processes on the synthetic networks compared to the references. Each figure displays five strategies that are arranged in rows. For the site percolation, we have the random attack in the first row followed by node arrangements according to the decreasing and increasing closeness centrality. The two last rows display the strategies based on the local degrees of the nodes. Similarly, in the bond percolation, the first row shows the random attack, and the next two rows apply to the ordering of the edges by degrees of their ends, i.e., the decreasing minimum end degree and the decreasing maximum end degree. In the last two rows, the multiplicity of the edges determines the sequence of their removal.

To catch a glimpse from the global and local point of view on the percolation process, we consider the following statistics. In each row, the first plot shows the percolation process on the entire multiplex whereas the subsequent three plots compare the resilience of the layers. The average, minimum, and maximum size of the largest component over all layers are computed and iteratively updated after a node or edge has been removed. In every plot, the relation of the percentage size of the largest connected component (*y*-axis) to the percentage of removed items (*x*-axis) is captured. The curves of the synthetic models represent average values over 100 synthetic multiplexes.

Let us first consider the plots concerning the percolation on the multiplex (i.e., the first column of plots in each figure). Looking at the site percolation results on both the EATN and the USATN, there is evidence of negligible differences in the effects on both the models and the references when using a random attack or an attack based on an increasing-order criterion. When applying the strategies based on decreasing-order criteria, in the EATN scenario, the three synthetic models tend to diverge over mid-regimes, with STARGEN showing similar resilience as the reference network, whereas BINBALL and ANGEL networks are less comparable to the EATN as their resilience is higher than the reference. In the USATN scenario, the three models tend to diverge up to high regimes in some cases, with BINBALL again being less comparable to the resilience of USATN, and ANGEL and STARGEN behaving similarly to the reference in this respect. Overall, the synthetic models exhibit a stronger resilience, which can generally be seen as an advantage when simulating ATNs. The results on bond percolation also reveal a fairly homogeneous behavior for all synthetic networks and their respective references across all regimes of the removal fraction, with the exception of BINBALL showing better resilience with respect to 50% and 75% of removed nodes.

Now, we consider the plots showing the percolation effects on layers, i.e., the average, minimum, and maximum size of the largest component over all layers shown in the second, third, and fourth columns of plots in each figure. Concerning the site percolation results, we observe from the ‘mean’ plots that all models tend to be comparably or less resilient than the EATN or the USATN when using a random or an increasing-order criterion strategy. However, the opposite tends to occur when using a decreasing-order criterion. Looking at the ‘max’ plots, the STARGEN resp. ANGEL resilience tends to be the closest to the EATN when using an increasing- resp. decreasing-order criterion; for the USATN, ANGEL is the closest to this reference regardless of the criterion. BINBALL tends to be more resilient than all other networks, synthetic and real, only for the ‘min’ case with decreasing-order criterion and only over low-mid regimes. Apart from that, it generally remains the less preferred model in terms of resilience.

As for the bond percolation results, BINBALL resilience is generally lower than all other models including both references, at least when measuring the ‘mean’ and ‘max’ size of the largest component over all layers. Also, ANGEL and STARGEN seem to have a similar impact, with the former better on ‘mean’ plots over all regimes, and slightly worse over some restricted mid or mid-high regimes on ‘max’ plots. For ‘min’ plots, ANGEL again outperforms the other models with respect to the EATN.

### Validation against the network structure

When further evaluating the resilience of the multiplex networks, we take into account variations in the layer structure. Indeed, the airline networks we analyze consist of layers with hub-spoke or point-to-point structures, and it might be intuitive that point-to-point formations must be more resilient against local attacks in comparison to those centralized around hubs. To validate this conjecture, we will refer to the ANGEL model, as it is the only synthetic model that is able to replicate layers with point-to-point structures alongside hub-spoke formations, the latter being defaults in STARGEN and BINBALL.

**Fig 24:**
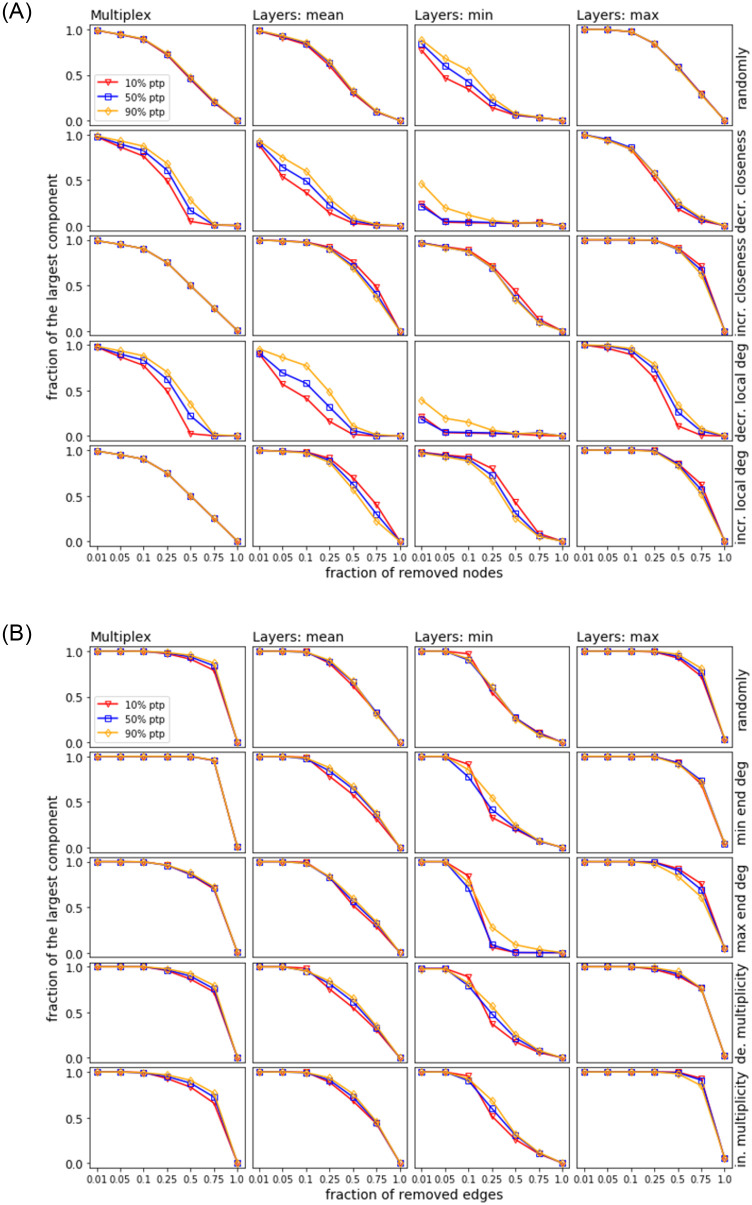
Site (top) and bond (bottom) percolation. Synthetic networks based on the EATN input data.

**Fig 25:**
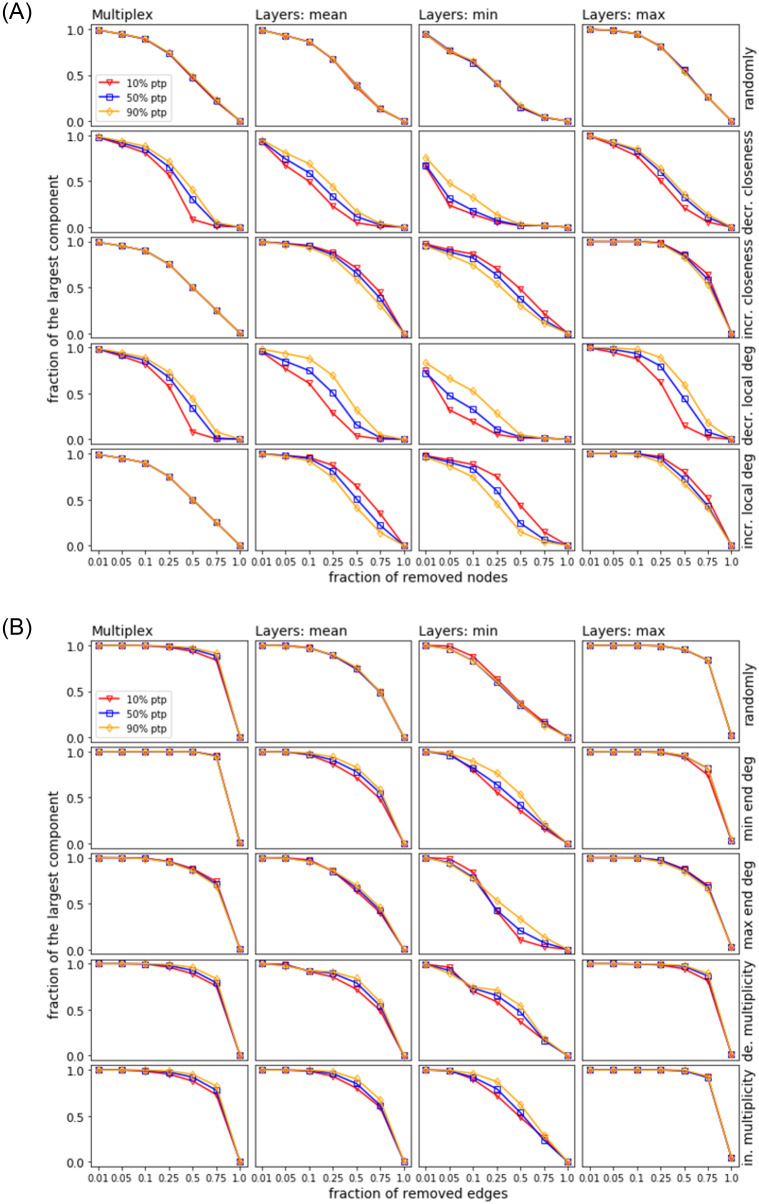
Site (top) and bond (bottom) percolation. Synthetic networks based on the USATN input data.

In Figs 24 and 25, we consider the same strategies and their representation as in the previous section. For each strategy (i.e., row in each plot), 100 samples of ANGEL are generated with the fixed input data as used for mimicking the reference network (i.e., # nodes, # edges, # layers, and distributions *P*_*edgeL*_, *P*_*nodeL*_, *P*_*layerN*_) except for the percentage of layers building a point-to-point or a hub-spoke structure. The thresholds used for the percentage of the point-to-point layers are 10%, 50%, and 90%, and the leftover corresponds to the hub-spoke layers. In this setting, the 10% threshold is the reference as it is the proportion of the point-to-point layers in the EATN and the USATN and also the default in the ANGEL model.

At a first glance, we observe from every plot in the figures a trend whereby higher percentages of point-to-point layers correspond to better resilience of the network, although significant differences in the percolation impact cannot be always detected. More specifically, for both the EATN and USATN scenarios, the above trend is more frequently observed for decreasing-order-based strategies in site percolation, while differences tend to be negligible over all strategies for bond percolation (with some deviations around mid regimes for the ‘min’ plots). Moreover, for site percolation, we observe an opposite yet less evident trend for increasing-order-based strategies. This can be explained since by removing nodes with lower closeness or local degree first, the hubs remain to the end and substantially determine the largest component, thus higher resilience is observed for lower percentages of point-to-point layers.

The above results confirm our expectations, although in many cases we can report null or small difference in the resilience behavior when using 50% (or even the default 10%) of point-to-point layers rather than the highest 90% of such layers. This suggests that networks generated by the ANGEL model can be equally resilient regardless of a particular setting of the point-to-point layer proportion, which is a unique feature of the ANGEL model against the competing ones.

## Spectral analysis

In this section, we analyze spectral and eigenfunction properties of the BINBALL, STARGEN and ANGEL models, and contrast them with the properties of our evaluation real-world ATNs, *i.e*., the EATN and the USATN. To this purpose, we resort to *Random Matrix Theory* (RMT) modeling.

### RMT models and measures

RMT has numerous applications in many different fields, from condensed matter physics to financial markets (*e.g*., [51]). In the case of complex networks, the use of RMT techniques might reveal universal properties. Among several studies available in the literature we can mention, as examples, that (1) the nearest-neighbor spacing distribution *P*(*s*) of the eigenvalues of the adjacency matrices of various network models follow Gaussian Orthogonal Ensemble (GOE) statistics [52]; (2) the *P*(*s*) and the entropic eigenfunction localization lengths of the adjacency matrices of Erdös-Rényi networks are universal for fixed average degrees [53]; (3) spectral and eigenfunction properties of multilayer networks [54], random rectangular graphs [55], and bipartite graphs [56] are universal for properly-defined scaling variables; and (4) RMT-based scaling analysis allows to predict the performance of network discovery algorithms [57].

In light of the above motivations, here we also use RMT modeling to analyze spectral and eigenfunction properties of heuristic and synthetic ATNs. Moreover, we consider an important modification to the standard adjacency matrix definition: We consider weights for vertices and edges in the layers of the ATNs studied here. Our main motivation to include weights, particularly random weights (*i.e*., statistically independent random variables drawn from a normal distribution with zero mean and variance one), is to retrieve well-known random matrices in the appropriate limits to use RMT results as a reference, see for instance [53–56]. With this prescription, the adjacency matrix of a completely disconnected network becomes a diagonal random matrix, known in RMT as the Poisson limit, whereas a member of the GOE is recovered for a fully connected network.

The adjacency matrix of the multiplex networks we consider here is a block matrix structured as follows. All blocks are *n* × *n* matrices, *n* being number of nodes of the multiplex. There are *l* × *l* blocks, *l* being the number of layers in the multiplex. Let *A*_*ij*_, *i*, *j* ∈ {1, …, *l*}, correspond to the block matrix in row *i* and column *j*. A diagonal block, *A*_*ii*_, *i* ∈ {1, …, *l*}, corresponds to the adjacency matrix of the *i*-th layer. *A*_*ij*_ = *A*_*ji*_, *i*, *j* ∈ {1, …, *l*}, *i* ≠ *j* are the so called *quasi-identity* matrices: we set 1 on the diagonal entry *a*_*kk*_ if node *k* belongs to both layers *i* and *j*. All other entries are zero. Finally, we replace all diagonal entries of the block adjacency matrix with independent Gaussian variables with zero mean and variance equal to 2. Clearly, since the adjacency matrices of the ATNs studied here are very sparse, we expect to observe a scenario close to the Poisson limit.

We use exact numerical diagonalization to obtain the eigenvalues λ_*n*_ and eigenfunctions Ψ^*n*^ (*n* = 1…*N*) of the adjacency matrices of size *N* = *n* × *l* of the ATNs. Specifically, in our study we use the nearest-neighbor spacings *s*, the ratios of consecutive level spacings *r*, and the entropic eigenfunction localization lengths *ℓ* to characterize spectral and eigenfunction properties of the adjacency matrices of weighted ATNs. We define *s*, *r*, and *ℓ* as follows.

Let λ_*n*_ be a set of ordered eigenvalues, then the corresponding nearest-neighbor spacings *s*_*n*_ and the ratios *r*_*n*_ are

$$\begin{array}{c} s_{n}=\frac{\lambda_{n+1}-\lambda_{n}}{\Delta }\;\text{and}\;r_{n}=\frac{\text{min}(s_{n},s_{n-1})}{\text{max}(s_{n},s_{n-1})}\;, \end{array}$$ (3)

respectively, where Δ is the mean eigenvalue density and *r* ∈ [0, 1]. The probability distribution functions of *s* and *r* in the Poisson limit, that we will use below as a reference, are as follows [58]:

$$\begin{array}{c} P_{\text{P}}\left(s\right)=\text{exp}\left(-s\right)\;\text{and}\;P_{\text{P}}\left(r\right)=\frac{2}{{\left(1+r\right)}^{2}}\;, \end{array}$$ (4)

respectively. It is important to stress that *P*(*s*) is already a well accepted quantity to measure the degree of disorder in complex networks, however, the use of *P*(*r*) is relatively recent; see an example in [59]. The entropic eigenfunction localization length of the eigenfunction Ψ^*n*^ is given as [53]:

$$\begin{array}{c} \ell_{n}=N\;\text{exp}[-(S_{\text{GOE}}-S_{n})]\;, \end{array}$$ (5)

where *S*_*n*_ is the Shannon entropy of Ψ^*n*^, defined as $S_{n}=-\sum_{m=1}^{N}{\left(\Psi_{m}^{n}\right)}^{2}\text{ln}{\left(\Psi_{m}^{n}\right)}^{2}$. In (5), *S*_GOE_ ≈ ln(*N*/2.07) is the entropy of a random eigenfunction with Gaussian distributed amplitudes. With this definition, the eigenfunctions of the adjacency matrices of a completely disconnected network have only one non-vanishing component with magnitude equal to one giving *S* = 0 and *ℓ* ≈ 2.07 ∼ 1. On the other hand, for a fully connected network, *S* ≈ *S*_GOE_ and the fully chaotic eigenfunctions extend over the *N* available vertices in the network, *i.e*., *ℓ* ≈ *N*. This measure provides the number of principal components of an eigenfunction in a given basis; *i.e*., *ℓ* ∈ [2.07, *N*] measures the extension of eigenfunctions.

### Validation against the EATN

**Fig 26:**
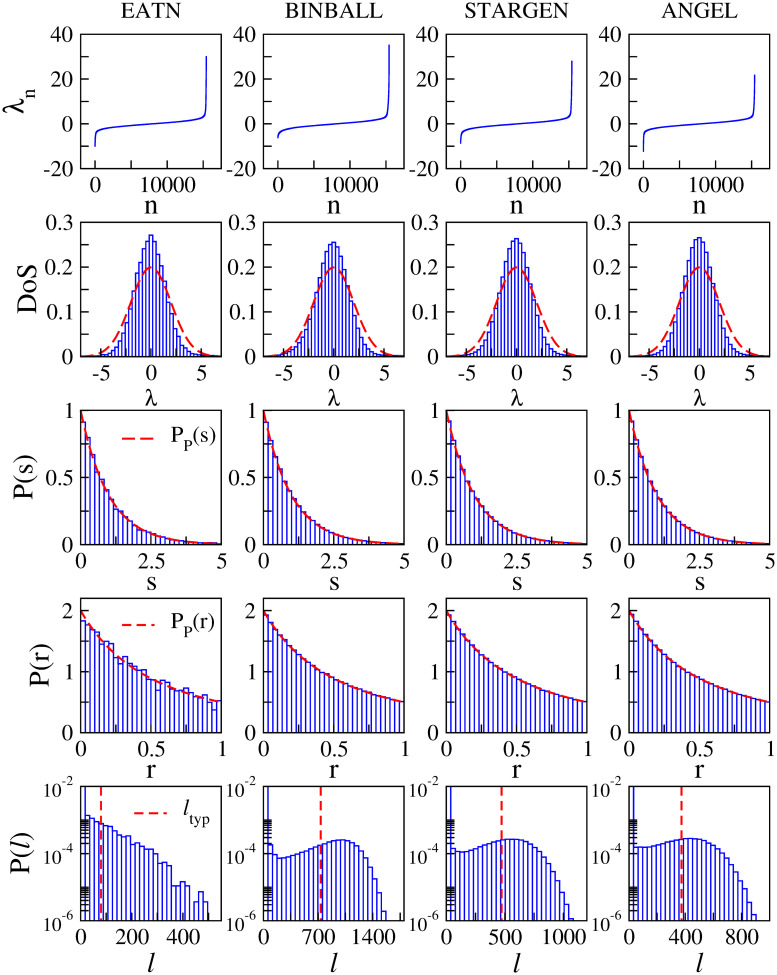
Spectral and eigenfunction properties. The EATN versus syn. models.

In Fig 26 we show spectral and eigenfunction properties of the EATN (first column) and of the corresponding synthetic models (BINBALL, STARGEN and ANGEL in second, third and fourth columns, respectively). Specifically we report: (first row) a single spectrum λ_*n*_; (second row) the density of eigenvalues or density of states, DoS; (third row) *P*(*s*); (fourth row) *P*(*r*); and (fifth row) the probability distribution of the entropic eigenfunction localization lengths, *P*(*ℓ*). We note that for the EATN all distributions (DoS, *P*(*s*), *P*(*r*), and *P*(*ℓ*)) are computed from a single randomly-weighted network; while in the case of the synthetic models all distributions are constructed from 100 random networks. Also, it is important to mention that *P*(*s*), *P*(*r*), and *P*(*ℓ*) were computed from 50% of the states located at the center of the spectra, where the states are expected to be equivalent.

From this figure we can see that none of the spectral quantities shown (λ_*n*_, DoS, *P*(*s*) nor *P*(*r*)) can distinguish between different ATNs. Moreover, while the DoS departs slightly from a Gaussian function (expected in the Poisson limit) both the *P*(*s*) and *P*(*r*) match the predictions corresponding to the Poisson limit; see Eq (4), also shown in red dashed lines.

Additionally, in Table 7 we report the values of 〈*r*〉, that indeed are very close to 〈*r*〉_P_ ≈ 0.38629 (reported from numerical simulations of diagonal random matrices [58]). This means that the EATN, as well as the corresponding synthetic models, show the properties of almost-diagonal random matrices; that is, all their eigenfunctions should be strongly localized. In the case of the EATN this is confirmed by the *P*(*ℓ*) which has a huge peak at *ℓ* = 2.07 and decreases in and exponential-like way for increasing *ℓ*. However, the *P*(*ℓ*) for the synthetic models presents an unexpected characteristic, that is, well defined local maxima for given *ℓ* ≫ 2.07. This local maxima in the *P*(*ℓ*) of the synthetic models reveals the existence of a well defined non-negligible set of extended eigenfunctions. Now, in order to roughly characterize the extension of those eigenfunctions we compute the average value of *ℓ*, 〈*ℓ*〉, but excluding the values of *ℓ* that contribute to the peak of *P*(*ℓ*) at *ℓ* ≈ 2.07. The obtained values of 〈*ℓ*〉 (shown as vertical red-dashed lines in the lower panels of Fig 26) are reported in Table 7, also for the EATN. Finally, it is relevant to remark that, even with the presence of extended eigenfunctions, ANGEL provides the best approach to the EATN in the case of eigenfunction properties.

**Table 7:** Spectral and eigenfunction properties. The EATN versus syn. models.

	EATN	BINBALL	STARGEN	ANGEL	Poisson limit
〈*r*〉	0.38597	0.38702	0.38604	0.3859	0.38629
〈*ℓ*〉	79.579	733.5	472.3	373.4	2.07

### Validation against the USATN

**Fig 27:**
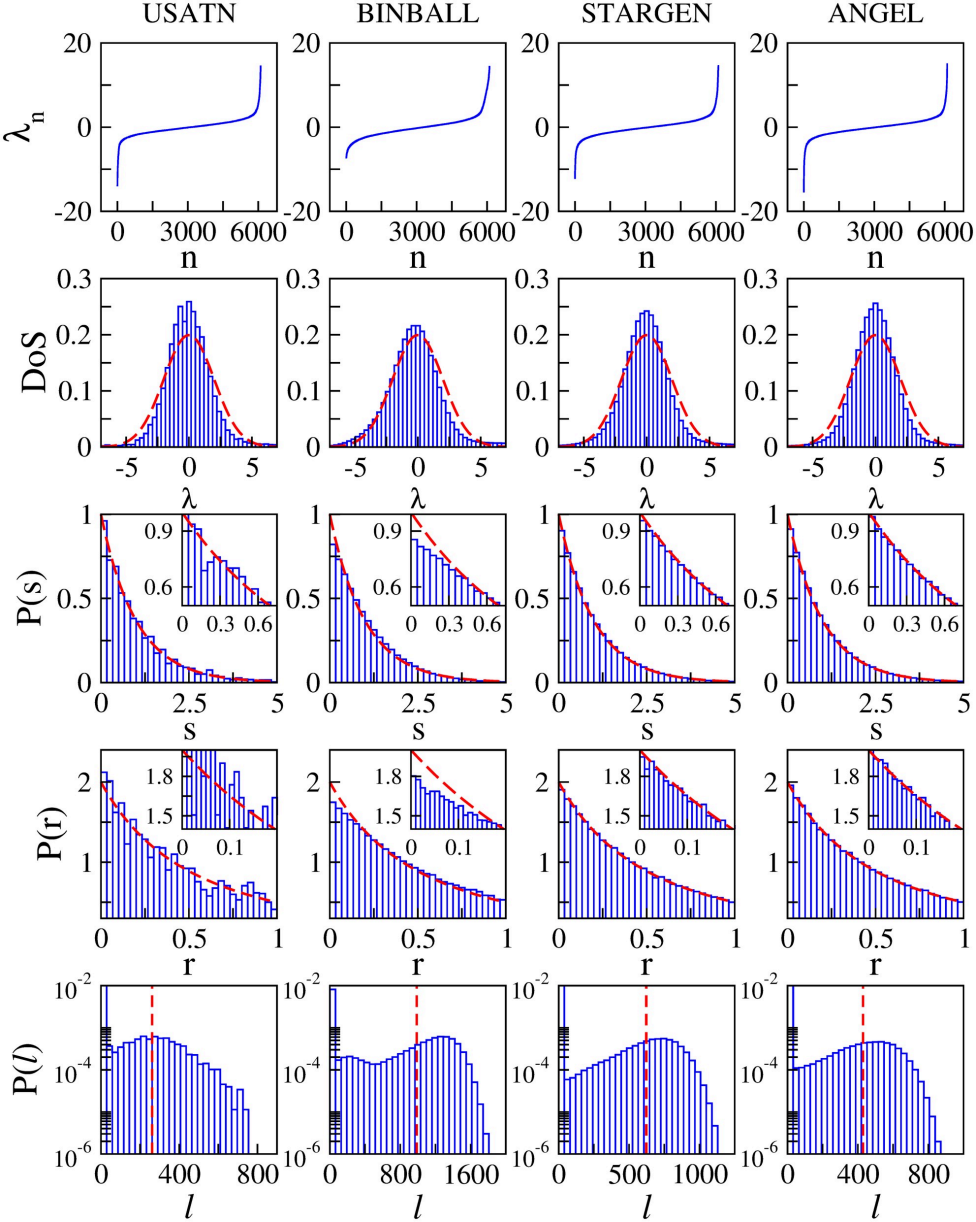
Spectral and eigenfunction properties. The USATN versus syn. models.

In Fig 27, we show spectral and eigenfunction properties of the USATN. We used the same coding as in Fig 26. We also report the values of 〈*r*〉 and 〈*ℓ*〉 obtained for the USATN and the corresponding synthetic models in Table 8.

**Table 8:** Spectral and eigenfunction properties. The USATN versus syn. models.

	USATN	BINBALL	STARGEN	ANGEL	Poisson limit
〈*r*〉	0.3831	0.39794	0.38771	0.38685	0.38629
*ℓ*_typ_	261.3	987.4	622.2	429.2	2.07

The situation we observe for the USATN is quite similar to the one reported for the EATN, with two noteworthy aspects that are reported as follows:

- Both *P*(*s*) and *P*(*r*) for BINBALL fall below the Poisson limit for small *s* and *r*, see the insets in the corresponding panels. While the *P*(*s*) and the *P*(*r*) for the USATN, STARGEN and ANGEL fall on top of *P*_P_(*s*) and *P*_P_(*r*), respectively. This means that STARGEN and ANGEL reproduce better than BINBALL the spectral properties of the USATN.
- The *P*(*ℓ*) of the USATN shows a maximum for *ℓ* ≫ 2.07, observed in the EATN analysis for the synthetic models only. Moreover, when comparing 〈*ℓ*〉, it is fair to say that ANGEL provides the best approach to the eigenfunction properties of USATN.

## Running times

**Fig 28:**
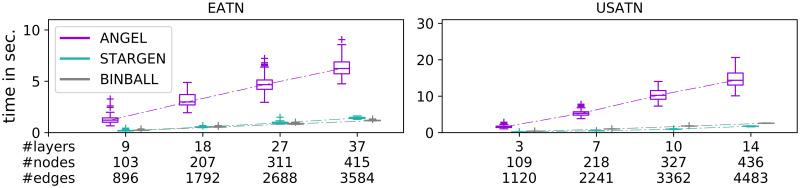
Running times of the generative algorithms when mimicking the EATN (left) and the USATN (right).

All three algorithms for synthetic creation of multiplexes were implemented in Python 3.6.0 and carried out on 2.2 GHz Intel Core i7, macOS 10.14.5, 8GB RAM. The running times of the routines broken down by the replicated reference are reported in Fig 28. To compare the sensitivity of the algorithms to the data volume, we scaled the original input data size (node, edge, and layer number) with the factors 0.25, 0.5, 0.75, and 1.0 respectively. The ticks on the *x*-axis correspond to these factors and are labeled with the corresponding number of layers, nodes, and edges.

As it can be seen from the plots corresponding to both reference networks, all three models exhibit running times that grow linearly with the increase in the data size. However, while STARGEN and BINBALL appear to be less sensitive to the data size due to their simpler methodology, ANGEL computing times show a relatively higher growth. Nonetheless, despite the higher complexity in the design compared to the other two models, the ANGEL algorithm is still efficient as it scales linearly with the data size.

It should be noted that the above results are consistent with the time computational complexity analysis of the three models. In fact, for both BINBALL and STARGEN, the main loop iterates over all *m* edges that are to be added to the multiplex, and in each pass, two vertices are randomly selected from one layer; therefore, both methods have a time cost of $O(m\cdot n_{max}^{L})$, where $n_{max}^{L}$ is the maximum number of vertices assigned to a layer. The running time of the ANGEL algorithm depends on the calculation of a minimum spanning tree on a complete graph spanned on all nodes in each layer of the hub-spoke structure. We observed that ${\left(n_{max}^{L}\right)}^{2}$ is O(m), *i.e*., ${\left(n_{max}^{L}\right)}^{2}=c\cdot m$, and *c* does not exceed the value 5 in the considered network data. Therefore, the running time can be estimated by $O(l\cdot m\cdot \text{log}\left(n_{max}^{L}\right))$, where *l* is the number of layers. This explains the moderate growth of the running times of ANGEL in relation to the increasing data size, in particular the number of edges in the multiplex.

## Discussion

In this section, we provide a summary of the main findings of the generative models for synthetic multilayer ATNs that we have considered in this work. Building upon our analysis in the previous sections, we have identified a number of features that are relevant to the three models, as reported in Table 9 that will be used as a guide to our discussion.

**Table 9:** Summary of features of the ATN synthetic models.

feature	BINBALL	STARGEN	ANGEL
*per-layer node distribution*	uniform	uniform	exponential
*per-layer edge distribution*	uniform	exponential	exponential
*per-node layer repetition distribution*	no	no	yes
*generated structures*	marginally hub-spoke	hub-spoke	hub-spoke & point-to-point
*incremental layer-network generation*	no	no	yes
*attraction of hub nodes*	weak	strong	strong
*non-hub repetition*	low	high	high
*spectral and eigenfunction properties replication of reference ATN*	weak	weak	strong
*network robustness*	less preferred	second best preferred	best preferred
*time complexity*	$O(m\cdot n_{max}^{L})$	$O(m\cdot n_{max}^{L})$	$O(l\cdot m\cdot \text{log}\left(n_{max}^{L}\right))$

The first three features in the table refer to the main model-parameters besides the three basic dimensions of the multilayer network (*i.e*., number of nodes, edges and layers), namely the distribution *P*_*nodeL*_ of the layer node-set size, the distribution *P*_*edgeL*_ of the layer edge-set size, and the distribution *P*_*layerN*_ of the per-node layer replication. In this regard, BINBALL can only produce network layers with similar sizes of both node and edge sets; this limitation is partially overcome by STARGEN as it can handle non-uniform edge counts of the layers. In ANGEL, this aspect is significantly enhanced as not only this model is able to control the repetition of nodes in the layers but also their overlapping. Overall, according to all the above parameters, ANGEL represents the most flexible model.

The above key advantage of ANGEL is further strengthened by its unique capability of controlling the formation of point-to-point structures in the layers alongside the hub-spoke structures, where the latter are also generally more exposed than in STARGEN and especially BINBALL. Moreover, this ANGEL’s feature is coupled with another distinguishing aspect, which enables a sort of incremental generation of the layer networks: indeed, unlike BINBALL and STARGEN, ANGEL is designed to generate layers separately from each other, which enables the model to mimic individual layers of the reference multilayer network.

In terms of network resilience, while STARGEN and ANGEL again outperform BINBALL—w.r.t. most of the attack strategies considered, under site and bond percolation scenarios—ANGEL can be equally resilient regardless of a particular setting of the point-to-point layer proportion, which is a unique feature of the ANGEL model against the competing ones. In addition, spectral analysis results based on RMT modeling w.r.t. both EATN and USATN modeling have shown that ANGEL is the best approach to characterize spectral and eigenfunction properties of the adjacency matrices of weighted ATNs.

In terms of efficiency, all methods scale linearly with the size of the network, with BINBALL and STARGEN having identical asymptotic time complexity, and ANGEL being sensitive to the number of layers of the network.

Despite some notable features shown by the generative models analyzed, particularly ANGEL, it should be noted that there are a number of limitations that encourage further research on synthetic modeling of ATNs.

For instance, STARGEN tends to be the closest to the reference in terms of edge-set size, but at the cost of a higher number of nodes per layer. ANGEL delivers much higher diversification in terms of node degrees and a better overall percentage of nodes shared among the layers than BINBALL and STARGEN, however the proportion of nodes that a synthetic layer has in common with others remains in a small range compared to the reference network layers. BINBALL replicas feature hub-spoke formations with very weak attraction by hubs, which is certainly not the case in ANGEL and STARGEN, however here, hub-spoke formations appear not as strong as in the reference networks. Moreover, although ANGEL focuses on structures of the layers in the multiplex (as suggested by its name, *i.e*., Air Network Generation Emphasizing Layers) and to replicate them it incorporates parameters to control node-set and edge-set sizes as well as the layer repetitions per node, this model still weakens when it comes to reproducing the interconnection between the layers. The problem lies in the difficulty of capturing this multidimensional relationship, and thus to measure and model it.

## Conclusions

Our presented study has focused on the opportunity of leveraging generative models conceived for the creation of synthetic multilayer ATNs in order to analyze the properties of real-world large-scale ATNs. To this end, we have thoroughly analyzed the state of the art for such models, namely BINBALL, STARGEN, and ANGEL, against two benchmark ATNs, *i.e*., the European and the U.S. ATNs. Our assessment concerned the ability of the three methods to be compared with the structural, spectral and resilience properties of the reference networks on both the global level of the multiplexes and the local level of the layers. We have also provided a summary of the key findings from our study to highlight the similarities and differences between the three models, as well as their limitations, both when compared with one another and with the reference networks.

We hope that our work can pave the way for further development of generative models for ATNs. Among the several directions that could be drawn, we raise the opportunity of incorporating side-information—in the form of attribute objects at node and/or edge level—as well as time-aware variables (*e.g*., flight departure and arrival times): indeed, while enriching the representation of an ATN and enabling the evolution over time of an ensemble of airline layers, these also lead synthetic generative models to deal with new challenges. Another interesting line of research would involve representation learning approaches and the exploitation of their knowledge patterns extracted from real-world ATNs into the design of a synthetic generative model for ATNs.

Finally, from an application perspective, we envisage the usefulness of synthetic ATN generative methods for simulating human mobility scenarios, also in critical situations like epidemic spread phenomena, so to help design appropriate intervention strategies and evaluate enhanced transport policies.
