# Correction: Structural and spectral properties of generative models for synthetic multilayer air transportation networks

**DOI:** 10.1371/journal.pone.0262383

**Published:** 2021-12-31

**Authors:** Marzena Fügenschuh, Ralucca Gera, José Antonio Méndez-Bermúdez, Andrea Tagarelli

In the Funding section, the following statement is missing: The views expressed in this document are those of the authors and do not reflect the official policy or position of the Department of Defense or the U.S. Government.
